# Doping strategies for small molecule organic hole-transport materials: impacts on perovskite solar cell performance and stability

**DOI:** 10.1039/c8sc05284k

**Published:** 2019-01-15

**Authors:** Tracy H. Schloemer, Jeffrey A. Christians, Joseph M. Luther, Alan Sellinger

**Affiliations:** a Department of Chemistry , Colorado School of Mines , Golden , CO , USA . Email: aselli@mines.edu; b Materials Science Program , Colorado School of Mines , Golden , CO , USA; c National Renewable Energy Laboratory , Chemistry and Nanoscience Center , Golden , CO , USA; d Hope College , Holland , MI , USA

## Abstract

Hybrid organic/inorganic perovskite solar cells (PSCs) have dramatically changed the landscape of the solar research community over the past decade, but >25 year stability is likely required if they are to make the same impact in commercial photovoltaics and power generation more broadly. While every layer of a PSC has been shown to impact its durability in power output, the hole-transport layer (HTL) is critical for several reasons: (1) it is in direct contact with the perovskite layer, (2) it often contains mobile ions, like Li^+^ – which in this case are hygroscopic, and (3) it usually has the lowest thermal stability of all layers in the stack. Therefore, HTL engineering is one method with a high return on investment for PSC stability and lifetime. Research has progressed in understanding design rules for small organic molecule hole-transport materials, yet, when implemented into devices, the same dopants, bis(trifluoromethane)sulfonimide lithium salt (LiTFSI) and tris(2-(1*H*-pyrazol-1-yl)-4-*tert*-butylpyridine)cobalt(iii) tri[bis(trifluoromethane)sulfonimide] (FK209), are nearly always required for improved charge-transport properties (*e.g.*, increased hole mobility and conductivity). The dopants are notable because they too have been shown to negatively impact PSC stability and lifetime. In response, new research has targeted alternative dopants to bypass these negative effects and provide greater functionality. In this review, we focus on dopant fundamentals, alternative doping strategies for organic small molecule HTL in PSC, and imminent research needs with regard to dopant development for the realization of reliable, long-lasting electricity generation *via* PSCs.

## Abstract 2

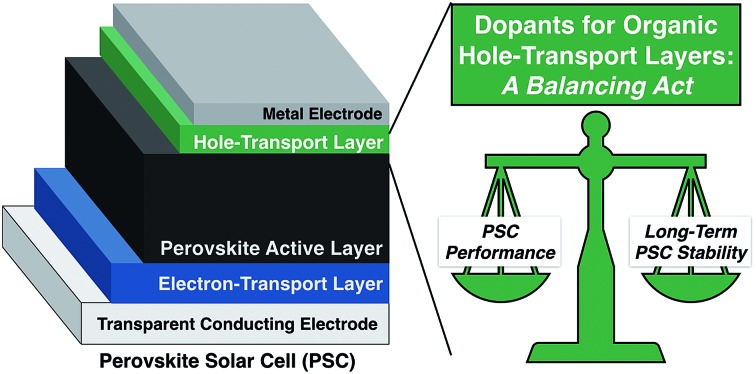
Dopants for small molecule-based organic hole-transport layers impact both perovskite solar cells initial performance and long-term stability.

## 1. Introduction

### 1.1 Perovskite solar cells

**Fig. 1:**
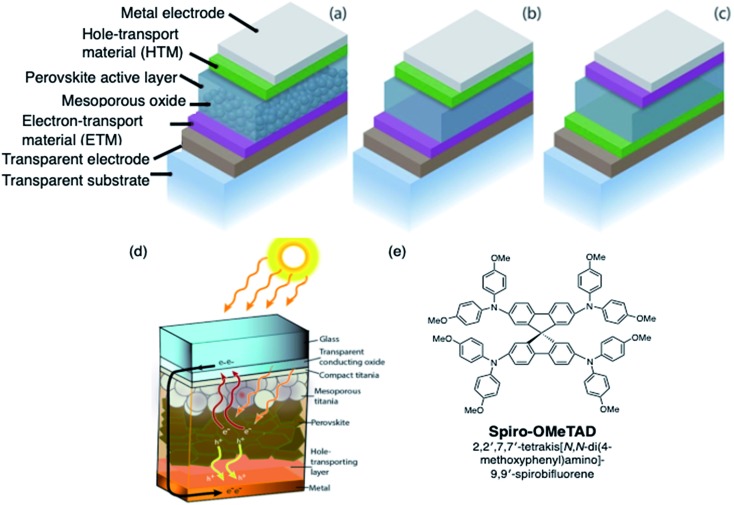
Schematic representation of different PVSC device architectures with the (a) mesoporous n–i–p configuration, (b) planar n–i–p configuration, and (c) planar-inverted (p–i–n) device stacks. Reprinted with permission from ref. 11. Copyright 2015 John Wiley and Sons. (d) Schematic diagram of a typical perovskite solar cell with the perovskite layer embedded between low and high workfunction layers. Reprinted with permission from ref. 6. Copyright 2017 American Chemical Society. (e) Chemical structure of spiro-OMeTAD.

With global energy demand projected to grow nearly 50% by 2040,^1^ it is imperative to divest from traditional greenhouse gas-based power production toward renewable energy sources such as solar. Perovskite solar cells (PSCs) represent the type of breakthrough solar technology to make solar cells and clean energy more ubiquitous. Hybrid organic/inorganic PSCs utilize an ABX_3_ perovskite crystal structure active layer, typically prepared from methylammonium (MA), formamidinium (FA), and/or cesium cations, lead and/or tin, and halide(s). The result is a highly absorbing semiconductor material with excellent semiconducting properties, such as high absorption coefficient, high carrier mobility and lifetime, and long carrier diffusion length.^2^–^5^ Common device architectures and device operation are summarized in Fig. 1. Briefly, since halide perovskites are relatively high dielectric materials, free electrons and holes, rather than bound excitons, are generated in the active layer as light is irradiated on the solar cell.^6^ The electrons flow through the electron transport layer (ETL) towards the anode, while holes migrate towards the cathode through the hole transport layer (HTL). The energetics and mobilities of each layer must be highly aligned and balanced to minimize charge accumulation and prevent recombination of positive charge carriers, or holes, and electrons. For instance, the HTL provides a pathway for both: hole conduction and electron blocking, which results in reduced recombination and increased fill factor (FF) and power conversion efficiency (PCE).^7^,^8^ Through extensive work in the latter half of the past decade,^9^–^13^ PSC efficiencies now rival commercial photovoltaic materials,^14^–^16^ despite the fact that they are processed from solution.

Nevertheless, to realize commercialization, a number of barriers must be addressed that address the full lifecycle of this technology (*e.g.*, improving module efficiency, stability and lifetime, and disposal of lead and other heavy metals, *etc.*).^17^ For the purposes of this text, stability will be defined as a change in PCE over time with respect to specific set of conditions, while lifetime considers not only stability but also the total length of power generation.^17^,^18^ While tremendous gains have been made in a number of areas,^19^–^24^ since the HTL is generally on top of the perovskite layer it acts as a first line of defense to protect perovskite structural integrity and therefore HTL engineering has a tremendous impact on PSC stability and lifetime.

### 1.2 Hole-transport materials

The first breakthrough in PSC technology was the application of an amorphous solid state hole transport layer^25^,^26^ to replace iodide liquid electrolyte used in the earliest reports.^27^ With the application of the spiro-OMeTAD (2,2′,7,7′-tetrakis[*N*,*N*-di(4-methoxyphenyl)amino]-9,9′-spirobifluorene, Fig. 1) layer, the performance skyrocketed beyond 10% to the present day record of 23.3%.^14^ The doped spiro-OMeTAD layer is not given enough credit in the development of this technology: without this easy-to-process layer, perovskite solar cells may have never attracted so much attention.

A unique challenge in hybrid organic/inorganic PSCs is that the perovskite cannot withstand harsh processing upon deposition of a subsequent layer. For example, a number of inorganic materials^28^ (*e.g.*, copper iodide, nickel oxide, copper oxide) have been explored as replacement HTLs for PSC, and such materials could offer improved stability, but often require incompatible or high processing temperature, making oxide-based HTLs desirable only for devices with p–i–n architecture (which implies substrate/HTL/perovskite/ETL) and thus they are typically not processed on top of the fragile perovskite film.^29^–^32^ Inorganic copper thiocyanate, CuSCN,^33^ is one such HTL that can be used in the n–i–p (substrate/ETL/perovskite/HTL) architecture, but there are still fabrication challenges: performance is dependent upon a multi-step dynamic deposition on the perovskite film for good surface coverage and crystallization with minimal perovskite dissolution. Aside from specific cases, the greater majority of perovskite solar cell research, and especially record efficiency,^14^,^34^ has relied on an organic-based HTL PSC designs.

Organic polymers have been shown to be effective HTLs, like poly[bis(4-phenyl)(2,4,6-trimethylphenyl)amine] (PTAA), poly(3,4-ethylenedioxythiophene) polystyrene sulfonate (PEDOT:PSS), or poly(3-hexylthiophene) (P3HT), due to their relatively high intrinsic conductivities and mobilities.^19^,^35^–^40^ Organic polymers are solution-processable and demonstrate decent thermal stability and hydrophobicity (with the exception of PEDOT:PSS, which has been shown to be hydrophilic^39^,^41^), and a number of recent reports highlight progress with polymeric HTLs for stable PSCs.^13^,^24^,^39^,^42^–^53^ Batch-to-batch molecular weight variation can alter thermal, morphological, and optoelectronic properties and subsequent PSC performance.^54^ Maintaining batch-to-batch molecular weight consistency can significantly drive up cost,^55^ which may reduce the feasibility for integration into a price-competitive commercial product. For further discussion, we refer readers to discussions in ref. 39, 47, 48 and 50.

Small molecule-based HTLs, rather than organic polymers, are versatile with respect to property tuning (*e.g.*, energetic tuning, hydrophobicity, film morphology, *etc*.) and demonstrate high batch-to-batch consistency in not only synthesis and purification but device fabrication, as organic small molecule (OSM) HTLs are typically amorphous. The HTL nominally consists of not only the HTM but also a number of additives, and is the focus of this review. In the context of this review, HTL refers to the films of HTMs which also contain additives and dopants.

As mentioned above, spiro-OMeTAD (Fig. 1) is the most commonly used OSM HTM for PSCs, and was originally developed for solid-state dye-sensitized solar cells.^56^ While useful for fundamental research,^57^ the synthetic costs, poor thermal stability, and low intrinsic conductivity and mobility make it unsuitable for commercialization. Researchers world-wide have been exploring lower cost and more efficient alternatives, although improving beyond the performance of spiro-OMeTAD has proven challenging.^7^,^8^,^44^,^47^,^48^,^50^,^52^,^53^,^57^–^68^

**Fig. 2:**
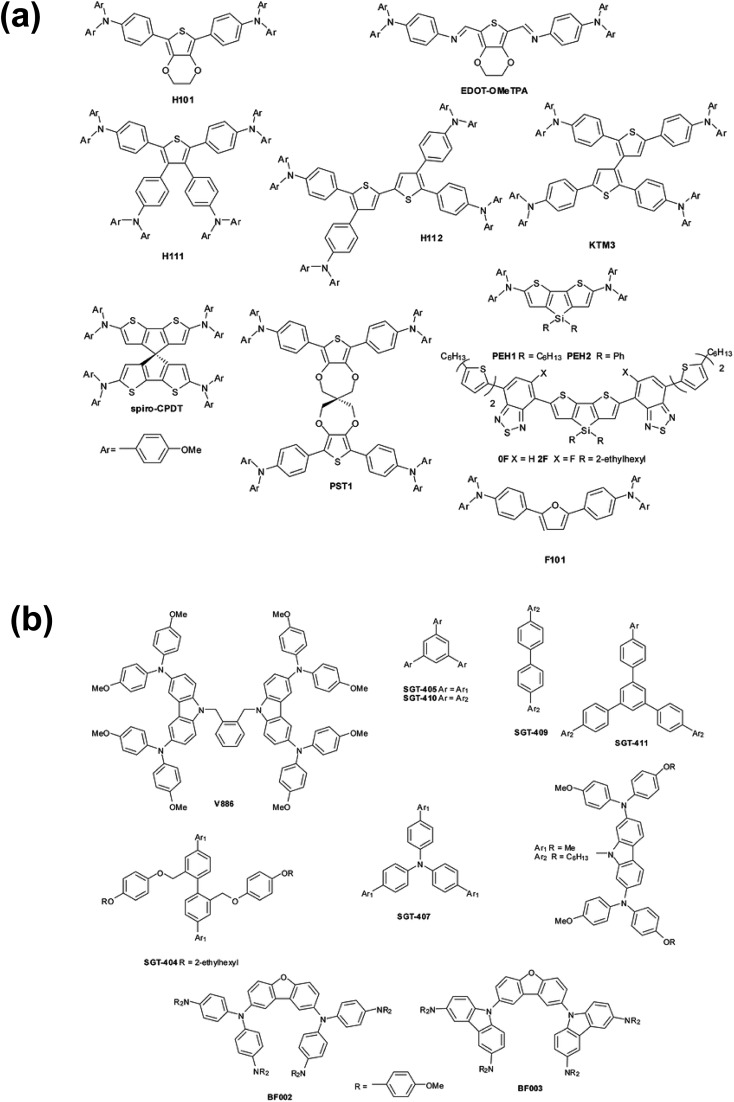
(a) Thiophene and furan-based HTMs, (b) dendrimer-like HTMs. Reproduced from ref. 48 with permission from The Royal Society of Chemistry.

OSM HTMs (Fig. 2) can be synthesized inexpensively and designed to possess most of the desirable properties, but unlike polymeric HTMs, the majority of new small molecule HTMs still require dopants to increase the low intrinsic conductivity and mobility. These dopants often contain hygroscopic and/or mobile ions, like Li^+^, and significantly decreases PSC stability with and without encapsulation (*vide infra*). Therefore, there remains an excellent opportunity to develop dopants which accomplish the goal of improving the HTL conductivity for efficient hole extraction, but offer improved hydrophilic properties, thermal stability, synthetic ease, *etc.*

Among the groups utilizing OSM HTLs, there have been two clear research avenues: dopant-free (*i.e.*, design a small molecule with sufficient conductivity/hole mobility without dopant^49^,^69^–^91^) and specialized dopant design research for improving HTLs with intrinsically poor conductivity/hole mobility. Because device efficiencies are generally higher with a doped HTL, as record device efficiencies have been achieved with a doped HTL,^14^,^34^ this review focuses upon the latter approach – alternative dopants for small molecule organic HTLs used in standard (n–i–p) device architecture PSCs. After discussing general dopant principles, alternative chemical dopants (ionic liquids, metal-based salts, oxidized radical cation salts, TCNQ derivatives) and relationship to PSC performance/stability will be discussed. A summary of reported PSC performance metrics are compiled chronologically in Table 1. We conclude with imminent research needs for HTL dopants for highly efficient and stable PSCs.

**Table 1:** Device performance with alternative HTL dopants as compared to reported control(s) outlined chronologically

Entry	Year	HTM	Dopant	Active layer/ETL	Fabrication method	*J*_sc_ (mA cm^–2^)	*V*_oc_ (V)	FF (%)	PCE (%)	Stability assessment	Ref.
1	2013	Spiro-OMeTAD	HTFSI	MAPbI_3–*x*_Cl_*x*_/c-TiO_2_	Solution	18.32	1.08	0.60	11.87	X	129
		Spiro-OMeTAD	LiTFSI + *t*BP			18.29	1.06	0.56	10.86	X	
2	2014	Spiro-OMeTAD	BuPyIm-TFSI	MAPbI_3–*x*_Cl_*x*_/m-TiO_2_/c-TiO_2_	Solution	16.26 ± 0.30	0.87 ± 0.04	0.56 ± 0.03	7.91 ± 0.30	X	133
		Spiro-OMeTAD	LiTFSI + *t*BP			15.56 ± 0.50	0.91 ± 0.05	0.57 ± 0.05	8.16 ± 0.25	X	
3	2014	Spiro-OMeTAD	Spiro(TFSI)_2_ + *t*BP	MAPbI_3_/m-TiO_2_/c-TiO_2_	Solution – N_2_ atmosphere, devices never exposed to air before testing	11.69	0.89	57	5.93	∼98% PCE retained (after air exposure and return to N_2_ atmosphere over 10 min illumination)	98
		Spiro-OMeTAD	LiTFSI + *t*BP	MAPbI_3_/m-TiO_2_/c-TiO_2_	Solution – N_2_ atmosphere, devices never exposed to air before testing	0.00217	0.99	37	0.000582	<90% PCE retained	
4	2014	Spiro-OMeTAD	AgTFSI + *t*BP	MAPbI_3–*x*_Cl_*x*_/Al_2_O_3_/c-TiO_2_	Solution	19.0 ± 2.21	0.90 ± 0.02	57 ± 6	11.2 ± 0.8	Improved stability with DSSC over 120 days; not reported for perovskite devices	161
		Spiro-OMeTAD	LiTFSI + *t*BP			18.2 ± 1.93	0.90 ± 0.02	48 ± 7	9.2 ± 0.9		
5	2014	Spiro-OMeTAD	IrCp*Cl(PyPyz)[TFSI] + LiTFSI + *t*BP	MAPbI_3–*x*_Cl_*x*_/bl-TiO_2_	Solution	15.90	1.064	64	10.8	96% PCE retained (3 months, ambient atmosphere, dark)	153
			LiTFSI + *t*BP			15.29	1.000	60	9.20	∼77% PCE retained	
6	2015	*po*-Spiro	CuPC 4.8 wt%	(FAPbI_3_)_0.85_(MAPbBr_3_)_0.15_/mp-TiO_2_/bl-TiO_2_	Solution	22.3	1.11	74.7	18.5	X	147
		*po*-Spiro	LiTFSI + FK209 + *t*BP			22.4	1.09	71.85	17.5	X	
7	2015	Spiro-OMeTAD	F4-TCNQ/pristine spiro-OMeTAD/DMC	MAPbI_3–*x*_Cl_*x*_/c-TiO_2_	Vacuum (fresh in air)	17.8	0.706	37.9	4.8	Steady PCE from 500–800 h, efficiency doubled as it aged in air (800 h, dark; measurement taken after 40 s under open circuit condition)	169
		Spiro-OMeTAD	LiTFSI + *t*BP	MAPbI_3–*x*_Cl_*x*_/c-TiO_2_	Solution (fresh in air)	23.1	0.967	60.3	13.5	∼50% PCE retained	
8		5,5′-Bis[4-trimethylstannyl-*N*,*N*-di(4-methoxyphenyl)aniline]-3,3′-diphenylsilylene-2,2′-bithiophene (PEH-2)	HTFSI + FK209 + *t*BP	MAPbI_3_/mp-TiO_2_/c-TiO_2_	Solution	19.4	0.97	72	13.5	65% maximum power output retained after 800 h (argon atmosphere, 45 °C, devices kept at maximum power point under 100 mW cm^–2^ light intensity with no light emission under 400 nm; 200–800 hours under light and inert gas conditions, but not always under applied bias voltage)	132
		Spiro-OMeTAD	HTFSI + FK209 + *t*BP			19.4	1.02	76	15.2	45% maximum power output retained after 800 h	
9	2016	Spiro-OMeTAD	CuSCN	MAPbI_3–*x*_Cl_*x*_/c-TiO_2_	Solution	22.01	1.06	77	18.02	∼80% PCE retained (unencapsulated, dark, in atmosphere, at room temperature, and the humidity in the range of 25–30%, 200 h)	148
		Spiro-OMeTAD	LiTFSI + *t*BP			20.12	1.06	69	14.82	∼35% PCE retained	
10	2016	EH44	EH44-ox + *t*BP	MAPbI_3–*x*_Cl_*x*_/Al_2_O_3_/c-TiO_2_	Solution	18.6	0.94	60	10.2	X	164
		Spiro-OMeTAD	Spiro-TFSI + *t*BP			18.1	0.89	56	10.3	X	
11	2016	Spiro-OMeTAD	F4TCNQ interlayer; LiTFSI/*t*Bp-doped Spiro-OMeTAD	MAPbI_3_/c-TiO_2_	Solution (fresh)	20.3 ± 0.8	1.06 ± 0.01	75.4 ± 2.7	16.4 ± 1.0	>60% PCE retained (ambient air dark storage, unencapsulated, 50–70% humidity, 960 h)	173
		Spiro-OMeTAD	LiTFSI + *t*BP		Solution (fresh)	19.4 ± 0.9	1.04 ± 0.03	69.9 ± 2.8	14.3 ± 0.9	40% PCE retained	
12	2016	Spiro-OMeTAD	F4TCNQ + LiTFSI	MAPbI_3–*x*_Cl_*x*_/c-TiO_2_	Solution	18.72	0.946	56.82	10.59	∼55% PCE retained (ambient air; 40–50% humidity)	172
			LiTFSI + *t*BP	MAPbI_3–*x*_Cl_*x*_/c-TiO_2_		19.70	0.974	64.86	12.66	∼10% PCE retained	
13	2016	VNPB	MoO_3_ on top of cross-linked VNPB	MAPbI_3_/PCBM/c-TiO_2_	Solution	18.6 ± 0.4	1.08 ± 0.02	76/71 ± 2.1(forward/reverse)	15.1 ± 0.6	95% PCE retained after 1 h at 100 °C on hot plate under N_2_, encapsulated	175
		Spiro-OMeTAD	LiTFSI + *t*BP			18.3 ± 0.4	1.06 ± 0.02	73/64 ± 3.0	14.0 ± 0.7	<70% PCE retained	
14	2017	Spiro-OMeTAD	H_3_PO_4_ + LiTFSI + FK209 + *t*BP	FA_0.85_Cs_0.15_PbI_3_/c-TiO_2_	Solution	21.88	1.06	0.76	17.6	∼85% PCE retained (shelf-life)	136
		Spiro-OMeTAD	LiTFSI + FK209 + *t*BP			21.61	1.02	0.69	15.2	∼85% PCE retained (shelf-life)	
15	2017	Spiro-OMeTAD	BCF + LiTFSI + *t*BP	MAPbI_3_/m-TiO_2_/c-TiO_2_	Solution	20.30	1.02	0.70	13.93	X	137
		Spiro-OMeTAD	LiTFSI + FK209 + *t*BP			19.18	0.99	0.69	11.73	X	
16	2017	Spiro-OMeTAD	Mo(tfd-COCF_3_)_3_	FA_0.85_MA_0.15_Pb(I_0.85_Br_0.15_)_3_/PC_60_BM/SnO_2_	Solution	21.6 ± 0.5	1.023 ± 0.040	70 ± 5	15.5 ± 1.5	∼70% PCE retained at SPO (unencapsulated, dark, 85 °C, 30–40% rh, 500 h)	140
		Spiro-OMeTAD	Mo(tfd-CO_2_Me)_3_			21.5 ± 0.4	1.039 ± 0.041	69 ± 5	15.4 ± 1.4	∼70% PCE retained at SPO	
		Spiro-OMeTAD	LiTFSI + FK209 + *t*BP			21.8 ± 0.3	1.121 ± 0.024	67 ± 3	16.4 ± 0.9	∼50% PCE retained at SPO	
17	2017	Spiro-OMeTAD	Cu(bpcm)_2_	(FAPbI_3_)_0.85_(MAPbBr_3_)_0.15_/TiO_2_	Solution	23.5 ± 0.22	1.09 ± 0.010	70.2 ± 1.20	17.9 ± 0.31	∼75% PCE retained (stability of PSCs under ambient conditions (humidity 30–40% and temperature 20–25 °C, devices were kept in dark condition after test, 20 days)	149
		Spiro-OMeTAD	FK209 + *t*BP			22.2 ± 0.88	1.04 ± 0.036	60.6 ± 3.12	14.0 ± 0.78	∼75% PCE retained	
18	2017	TaTm	F6TCNNQ	MAPbI_3_/C_60_/*N*^1^,*N*^4^-bis(tri-*p*-tolylphosphoranylidene)-benzene-1,4-diamine (PhIm)	Vacuum	20.28	1.115	79.8	18.0	∼75% PCE retained (constant illumination, ∼40 °C, unencapsulated, N_2_ atmosphere)	171
19	2017	Spiro-OMeTAD	Benzoyl peroxide + LiTFSI + *t*BP	MAPbI_3_/m-TiO_2_/c-TiO_2_	Solution	23.5 ± 0.1	0.993 ± 0.012	72 ± 2	16.79 ± 0.6	∼90% PCE retained (loss of 9.2% FF) (N_2_ atmosphere, 30 days)	154
		Spiro-OMeTAD	LiTFSI + *t*BP			21.8 ± 0.2	0.952 ± 0.050	65 ± 2	13.49 ± 0.9	∼70% PCE retained (loss of 24.3% FF)	
20	2017	Spiro-OMeTAD	H_4_PMo_11_V·*n*H_2_O + LiTFSI + *t*BP	MAPbI_3_/m-TiO_2_/c-TiO_2_	Solution	20.27	0.95	66	13.08	X	178
			LiTFSI + *t*BP			18.23	0.98	61	10.74	X	
21	2017	X44	TFSI^–^ incorporated into X44 salt	FA_0.85_MA_0.15_Pb(I_0.85_Br_0.15_)_3_)/m-TiO_2_/bl-TiO_2_	Solution	21.04	1.08	67	15.2	∼100% PCE retained – FF increased over time (<20% humidity, dark, unencapsulated, 15 days)	174
		Spiro-OMeTAD	None			18.39	1.08	38	7.5	∼95% PCE retained (<20% humidity, dark, unencapsulated, 15 days)	
22	2018	EH44	EH44-ox + *t*BP	(FA_0.76_MA_0.21_Cs_0.03_)_0.67_Pb(I_0.89_Br_0.11_)_2.56_/SnO_2_	Solution	22.35	1.088	67.9	16.52	88% avg PCE retained (1000 hours, 30 °C, 10–20% relative humidity, constant illumination, 510 Ω static load)	126
23	2018	Spiro-OMeTAD	FeCl_3_ + LiTFSI + *t*BP	Cs_*x*_(MA_0.17_FA_0.83_)_(1–*x*)_Pb(I_0.83_Br_0.17_)_3_//m-TiO_2_/c-TiO_2_	Solution	21.14 ± 0.42	1.11 ± 0.01	73.4 ± 1.1	17.2 ± 0.7	X	155
			FK209 + LiTFSI + *t*BP			20.57 ± 0.48	1.10 ± 0.01	71.8 ± 1.0	16.2 ± 0.6	X	
24	2018	Spiro-OMeTAD	Fe(ttb) (5 mol%) + LiTFSI + *t*BP	CsFAMARb “quadruple cation”/SnO_2_	Solution (perovskite), atomic layer deposited SnO_2_	21.7	1.20	74	19.2	X	156
		Spiro-OMeTAD	FK209 + LiTFSI + *t*BP		Solution (perovskite), atomic layer deposited SnO_2_	22.0	1.14	77	19.3	X	
25	2018	Spiro-OMeTAD	Li+@C60 + *t*BP	(FAPbI_3_)_0.85_ (MAPbBr_3_)_0.35_/C_60_	Solution	22.9	1.01	72	16.8	Devices functioned for ∼500 h, unencapsulated, ambient conditions, constant illumination	157
		Spiro-OMeTAD	LiTFSI + *t*BP			22.2	1.11	75	18.5	Devices functioned for ∼20 hours	
26	2018	Spiro-OMeTAD	Zn(TFSI)_2_ + FK209 + *t*BP	triple cation/mp-TiO_2_/cp-TiO_2_	Solution	23.90	1.15	78.4	21.52	100% PCE retained at 25 °C, 79% PCE retained at 50 °C (maximum power point, N_2_ atmosphere, 600 h)	150
		Spiro-OMeTAD	LiTFSI + FK209 + *t*BP			23.71	1.10	74.7	19.48	80% PCE retained at 25 °C, 45% PCE retained at 50 °C	
27	2018	Spiro-OMeTAD	BMPyTFSI (7.8 mol%)	(FAPbI_3_)_0.85_ (MAPbBr_3_)_0.35_/mp-TiO_2_/cp-TiO_2_	Solution	21.17	1.020	65.12	14.06	Up to 80% PCE retained over 200 days (ambient atmosphere, 50% relative humidity, unencapsulated, stored in dark)	135
			LiTFSI + FK209 + *t*BP			21.37	0.950	73.45	14.96	<50% PCE retained	
28	2018	Spiro-OMeTAD	JQ1 (9 mol%) +LiTFSI + *t*BP	Rb_0.05_Cs_0.05_FA_0.8_MA_0.1_Pb(I_0.85_Br_0.15_)_3_/mp-TiO_2_/cp-TiO_2_	Solution	22.8 ± 0.3	1.120 ± 0.006	75 ± 1	19.3 ± 0.2	X	152
		Spiro-OMeTAD	JQ3 (10 mol%) + LiTFSI + *t*BP	Rb_0.05_Cs_0.05_FA_0.8_MA_0.1_Pb(I_0.85_Br_0.15_)_3_/mp-TiO_2_/cp-TiO_2_		21.8 ± 0.3	1.020 ± 0.005	66 ± 3	15.5 ± 0.5	∼50% PCE retained over 1 week (∼175 h, ambient conditions, 50% relative humidity, 25 °C, dark)	
		Spiro-OMeTAD	JQ3 (10 mol%) + LiTFSI + *t*BP	1 nm Al_2_O_3_/Rb_0.05_Cs_0.05_FA_0.8_MA_0.1_Pb(I_0.85_Br_0.15_)_3_/mp-TiO_2_/cp-TiO_2_						∼94% PCE retained	
		Spiro-OMeTAD	LiTFSI + FK209 (3 mol%) + *t*BP			22.0 ± 0.5	1.090 ± 0.010	74 ± 2	18.0 ± 0.3	X	

## 2. Organic small-molecule doping principles and common practice

### 2.1 Doping in small-molecules

**Fig. 3:**
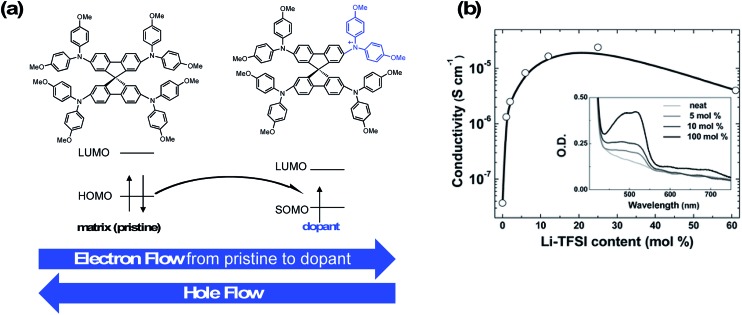
(a) Integer charge-transport mechanism with molecular p-type dopant. Adapted with permission from ref. 95. Copyright 2007 American Chemical Society. (b) Effective spiro-OMeTAD conductivity and UV-Vis absorption spectra (inset) as function of the Li-TFSI content. These films were left for 78 hours in air before measurement. The solid-line is simply to aid the eye. Reproduced from ref. 96 with permission from the PCCP Owner Societies.

A dopant is an impurity added to a bulk matrix (in this case a film of “pristine” organic small molecules) to alter its semiconductor properties.^92^ However, unlike in inorganic systems, the doping process in organic systems typically corresponds to chemical oxidation (specifically in p-type doping), and charge transport involves redox reactions.^93^Fig. 3 highlights a proposed mechanism for charge hopping in p-doped organic thin films.^94^,^95^ The singly occupied molecular orbital (SOMO) of the dopant (oxidized spiro-OMeTAD) is slightly deeper (lower in energy) than that of an electron in the HOMO of the pristine material; thus, there is a thermodynamic driver for integer charge hopping from pristine to dopant. In essence, when the dopant is reduced, it becomes chemically identical to the bulk matrix, and the pristine molecule becomes chemically identical to the dopant. This process repeats across the thin film as holes are accepted from the adjacent perovskite layer. For more detail on charge-transport mechanisms in organic systems, please refer to the discussions in Walzer, *et al.*^95^ and Lüssem, *et al.*^92^

Conductivity typically increases by multiple orders of magnitude as a function of dopant concentration as trap states are filled by the dopant, and then plateaus or decreases at higher dopant concentrations (Fig. 3). Dopant addition shifts the HTL Fermi level (*E*_F_) closer to the HOMO level of the pristine matrix,^97^ and the observed HOMO level of the HTL film to become slightly deeper (depending on doping concentration, ∼100–200 meV).^98^ Note that observed conductivity, mobility, and energy level shifts are dependent upon processing conditions (solution, vacuum), as morphology and impurity concentrations vary with each technique.^99^ Overall, the combination of increased conductivity, mobility, and energetic alignment are key for current matching among the HTL and perovskite during device operation. In turn, this allows for improved hole injection into the perovskite and reduced recombination at the interface, and are key screening tools for dopant assessment. Moreover, HTL series resistance decreases concomitant with an increase in conductivity due to doping^100^ which, in solar cells, leads to improved charge extraction and higher fill factor (FF).

### 2.2 Doping in PSC HTLs

**Fig. 4:**
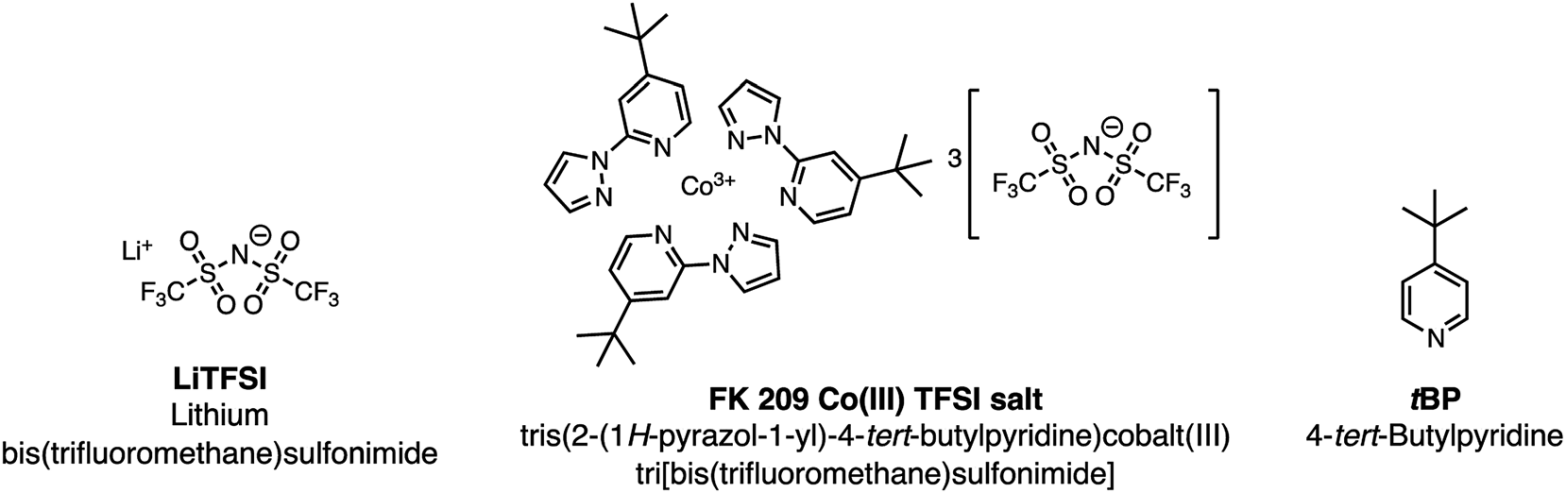
Chemical structures of common HTL dopants.

During conventional PSC fabrication, a solution of the pristine HTM material is doped *in situ*, and immediately spin-coated on top of the perovskite.^101^ The most widely used dopants for solution-based PSC fabrication, shown in Fig. 4, are lithium-based (*i.e.*, LiTFSI – bis(trifluoromethane)sulfonimide lithium salt) and cobalt-based dopants (*i.e.*, tris(2-(1*H*-pyrazol-1-yl)-4-*tert*-butylpyridine)cobalt(iii) tri[bis(trifluoromethane)sulfonimide] (FK209)), or a mixture of the two,^102^ along with 4-*tert*-butyl pyridine (*t*BP).

**Scheme 1:**
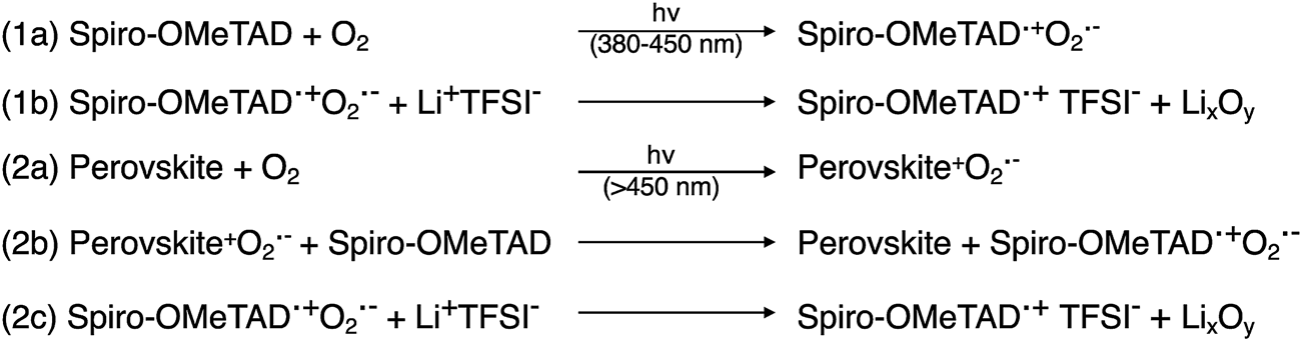
Spectrum-dependent generation of oxidized spiro-OMeTAD, at (1) short and (2) long-wavelength regimes. Adapted with permission from ref. 103. Copyright 2015 American Chemical Society.

For simplicity, the reactions involved in the doping process will be discussed with respect to spiro-OMeTAD as most studies investigate this system; however, it should be noted that many other HTL systems not based on spiro-OMeTAD utilize some or all of these standard dopants/additives. According to Abate *et al.*^96^ the pristine spiro-OMeTAD reacts with O_2_ to generate a weakly bound complex^99^ (Scheme 1) in the presence of light or heat after the thin film is exposed to air. However, the TFSI^–^ anion traps/stabilizes the radical cation, and the remaining metal cation (*e.g.*, Li^+^, Co^3+^) forms an oxide complex. Wang *et al.*^103^ further suggest a spectral-dependence of radical cation generation; specifically, in the presence of >450 nm light, radical cation generation can be initiated by the perovskite (Scheme 1). The film is considered to be p-doped once a critical concentration of radical cations is trapped. A non-trivial consequence of this *in situ* protocol is that it is challenging, if not impossible, to control how much oxidized HTM is trapped. With regard to nomenclature, in the broader literature, LiTFSI and FK209 are regarded as dopants. We will continue to refer to the radical cation-generating additives as dopants throughout this text. However, it is the radical cation species, and not the “dopant” (*i.e.*, LiTFSI, FK209), that is responsible for the improved HTL properties and ultimately dopes the HTL.

While doping of spiro-OMeTAD is largely dependent on ambient conditions, this conventional doping scheme is effective for decreasing HTL resistance. Compared to spiro-OMeTAD films which are undoped, the mobility^104^ and conductivity^102^ of doped spiro-OMeTAD films can be typically increased by at least an order of magnitude. Even though the charge-transport of spiro-OMeTAD is poor when compared to inorganic semiconductor materials, the thin nature of the HTL films in general still allows for the attainment of PV devices with FF > 80% – especially when doping with both LiTFSI and FK209.^102^ Nevertheless, the low hole mobility of spiro-OMeTAD does appear to limit device FF to some degree.^29^,^105^,^106^ This offers an opening for alternative HTL materials to prove advantageous as there is still significant room for PSCs in FF improvement as the maximum FF under the Shockley-Queisser limit framework is ∼90% for band gaps between 1.5–1.6 eV.^106^,^107^

**Fig. 5:**
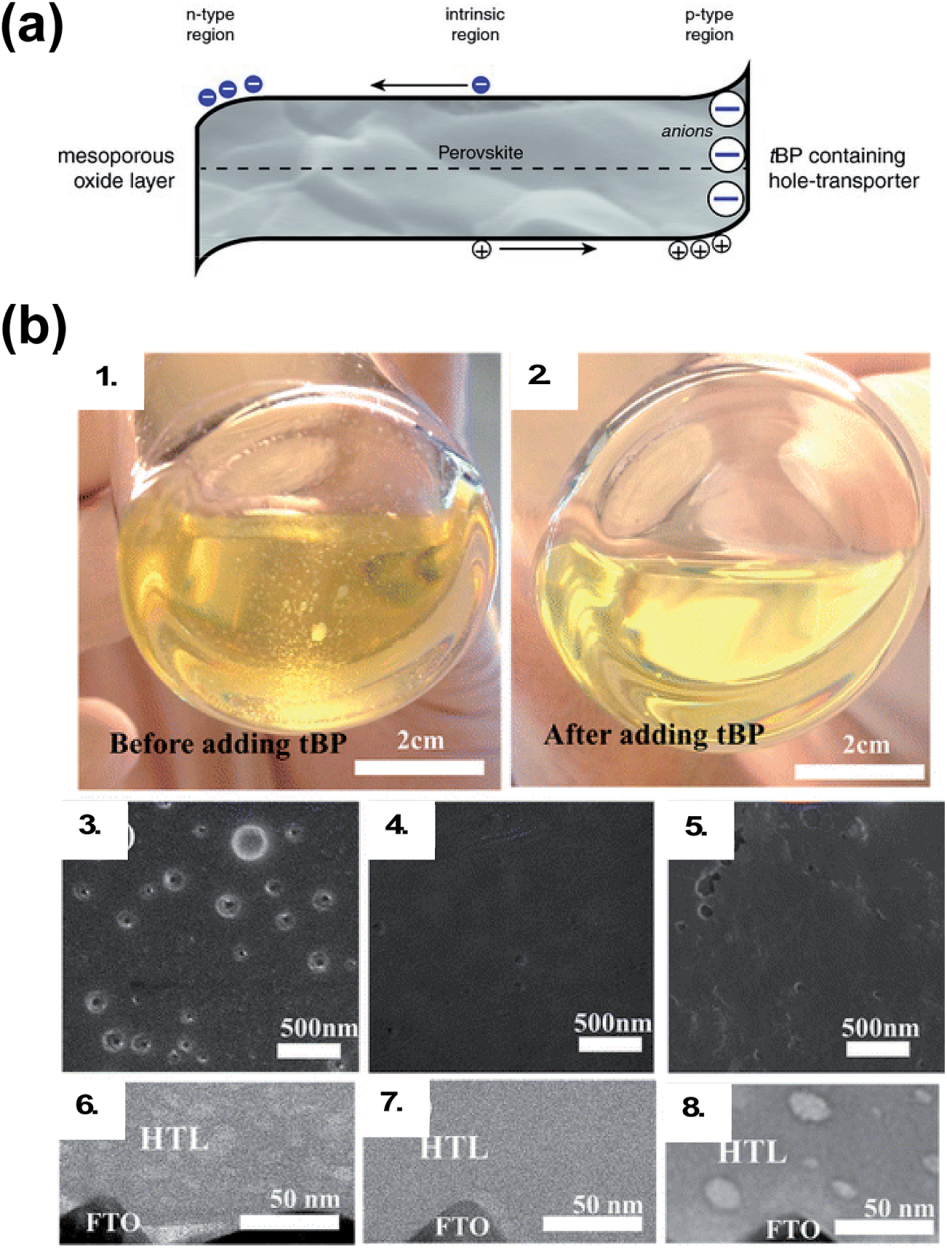
(a) Schematic of the proposed mechanism. The tBP contained in the hole-transporter effectively p-dopes the perovskite interface by creating a negative ionic charge layer at the interface. In order to maintain the charge neutrality of the crystal, mobile positive charges accumulate in this region effectively p-doping it. This results in band bending favoring the extraction of photogenerated holes over electrons. Reprinted with permission from ref. 113. Copyright 2016 John Wiley and Sons. (b) Photographs of HTL solution used for spin coating. (1) Before adding *t*BP and (2) after adding *t*BP. Top-view SEM images of the freshly prepared HTL (3) without *t*BP, (4) with *t*BP, and (5) with *t*BP after overnight vacuum treatment (10^–4^ Pa). Cross-section BF-TEM images of the freshly prepared HTL (6) without *t*BP, (7) with *t*BP, and (8) with *t*BP after overnight vacuum treatment (10^–4^ Pa). Reprinted with permission from ref. 111. Copyright 2016 American Chemical Society.

In addition to LiTFSI and FK209, 4-*tert*-butylpyridine (*t*BP) is a near-ubiquitous additive with chemical oxidants, yet it is unclear as to whether *t*BP imparts purely electronic or morphological benefits to the HTL. The main role of tBP is to prevent phase segregation of LiTFSI and spiro-OMeTAD, resulting in a homogeneous HTL.^108^ Recently, cation–pi interactions between *t*BP and Li^+^ ions were uncovered,^109^ which corroborates what is observed in practice: LiTFSI solubility can be modulated in the presence of *t*BP. Pinholes in HTL films have been shown to be related to the presence of small amounts of secondary solvents (*e.g.*, water, 2-methyl-2-butene, or amylene).^110^ Generally speaking, with *t*BP, HTL films show reduced pinhole formation (Fig. 5).^111^ With regard to electronic benefits to the HTL, *t*BP has been shown to suppress charge-recombination,^112^ and there is evidence that its addition makes the perovskite/HTL interface more hole-selective.^113^ To understand this effect, Habisreutinger *et al.* propose *t*BP is protonated by methylammonium (from the active layer), resulting in a slight negative charge at the perovskite interface and increased hole attraction from resulting energetic band bending (Fig. 5).^113^ In reality, *t*BP is likely involved in multiple processes.

**Fig. 6:**
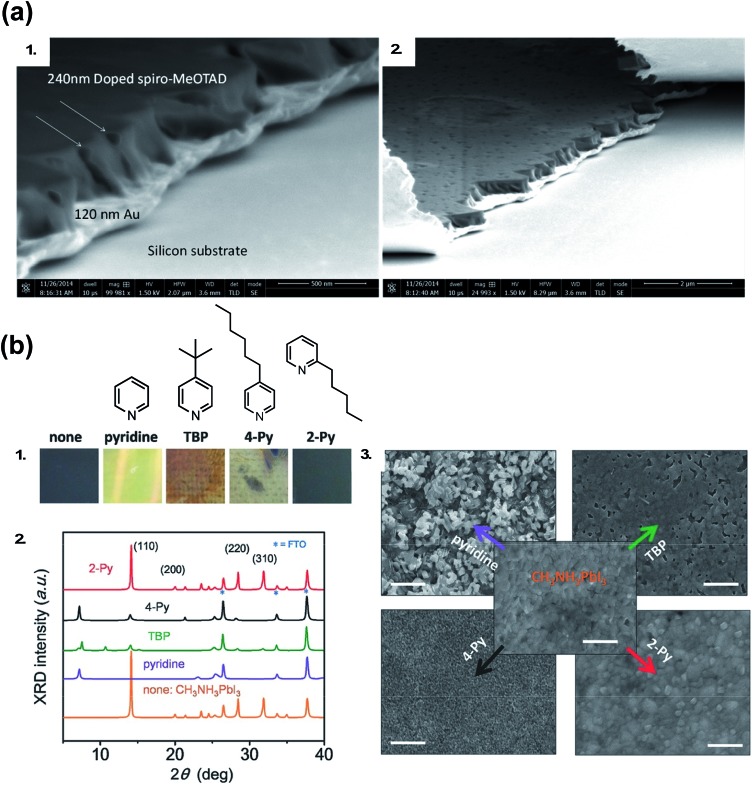
(a) Cross-sectional SEM of doped spiro-OMeTAD films on Au: (1) pinholes form channels across the doped spiro-OMeTAD film indicated with arrows in high magnification image, and (2) the pinholes observed from the top surface of the film and from the cross section. Reprinted with permission from ref. 116. Copyright 2015 American Chemical Society. (b) (1) Photos of perovskite films after separate treatments with one drop of pure pyridine additive. The spin-coating process was done in the N_2_-filled glove box. “none” denotes a pristine perovskite film. (2) The corresponding XRD patterns of the films after treatment with these additives. (3) SEM top-view images of perovskite films treated with these pyridine-based additives. Scale bars: 500 nm. Reprinted with permission from ref. 126. Copyright 2016 John Wiley and Sons.

Generally, dopants like LiTFSI and FK209 negatively impact PSC properties over time. While oxygen allows for radical cation generation upon LiTFSI addition to HTM, it is well known oxygen exposure is be detrimental to perovskite active layer (PAL) stability.^114^,^115^ With LiTFSI, there are a number of reported phase-segregation challenges:^116^,^117^ for instance, LiTFSI can migrate to the Au–HTL interface *via* film pinholes (Fig. 6). Li^+^ ions can migrate to the ETL through the active layer,^22^,^118^ which leads to device failure. Qi and coworkers propose water vapor is the main component in air responsible for enhanced conductivity in doped films, as it allows for LiTFSI to remain distributed throughout the HTL and the water is necessary for dopant function,^116^ but again, it is well known that water is detrimental to the perovskite active layer and device properties.^119^–^122^ LiTFSI also contributes to HTL delamination from the PAL.^123^ Little is known about direct effects of cobalt-based dopants on PSC stability,^124^ as devices typically do not achieve as high of FF and PCE without LiTFSI co-doping and are thus less well-studied.^102^

As researchers transition from cell- to module-level studies, scribe lines/module interconnects are susceptible to dopant-related corrosion.^125^ With respect to *t*BP, it has been shown to be volatile (Fig. 5)^111^ and dissolve perovskite in high concentrations *via* formation of a complex with PbI_2_ (Fig. 6).^126^ However, it is unclear how much *t*BP in the HTL impacts device stability,^127^ and changing pyridine functionalization (Fig. 6) or replacing with other salts^128^ can reduce corrosion. In the context of this review, *t*BP will not be regarded as a “dopant”; however, we certainly believe that further investigation into *t*BP with respect to mechanistics and stability is warranted. To summarize, commonly-employed dopants allow for record-efficiency devices but they also directly preclude device stability.

## 3. Alternative chemical dopant schemes

While comprehensive design criteria for HTL dopants in PSCs remain elusive, an ideal chemical dopant should

(1) Quantitatively and reproducibly generate highly stable radical cations at a reasonable rate for improved charge-transport (*i.e.*, dopant oxidation potential that readily leads to radical triarylamine generation for high HTL conductivity and mobility),

(2) generate inert byproducts or, if byproducts are inevitable, impart desirable properties (*i.e.*, increase hydrophobicity or thermal stability, reduce phase segregation),

(3) lead to highly efficient and stable PSC (*i.e.*, adequate HTL energetic alignment with PAL for hole extraction) while

(4) maintaining low cost (*i.e.*, not limited to inert conditions, few synthetic steps, tunable redox properties, limited need for polar co-solvent upon HTL deposition) to promote widespread accessability to researchers.

The ubiquity of LiTFSI-doped HTLs in PSC research can likely be attributed to some combination of historical precedent, broad commercial availability, simplicity of methods, and demonstrated high PSC performance. Nevertheless, LiTFSI fails multiple metrics when considered against the framework enumerated above. While LiTFSI reproducibly generates spiro-OMeTAD radical cations and does yield high performance PCSs, the reaction yield is not consistent due to the dependence on ambient conditions – one explanation for the need to optimize dopant concentration from lab to lab, assuming other factors are held relatively constant. More problematic, dopant byproducts, lithium oxides, increase HTL hydrophilicity, and lithium ions can migrate through the device stack.

Therefore, in this section, we will expand upon efforts which aim to fulfill each of these criterion towards universal dopant design criteria for HTMs in PSCs. Generally, they fall into the following categories: ionic liquids, Brønsted/Lewis acids, metal-based salts, oxidized radical cation salts, tetracyanoquinodimethane (TCNQ) derivatives, and other miscellaneous schemes.

### 3.1 Ionic liquids

**Fig. 7:**
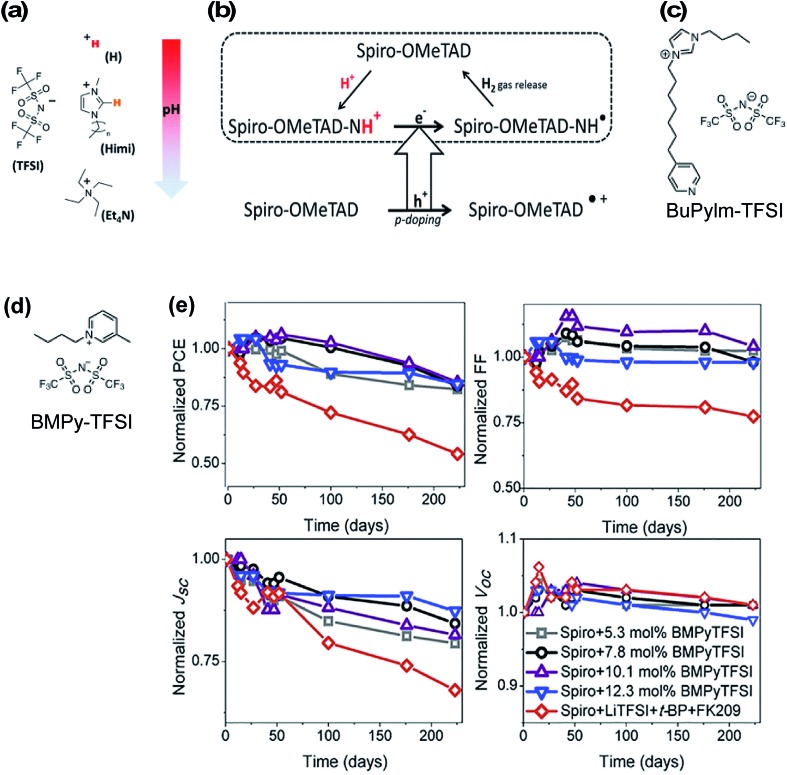
(a) Chemical structure of ionic liquids used in this study: H-TFSI and Himi-TFSI (*n* = 5 for the data reported in this work) are PILs, with H-TFSI more acidic (free proton in red) than Himi-TFSI (the most acidic proton in orange), and Et_4_N-TFSI as an aprotic ionic liquid. The pH scale may be considered as an indication of how strongly a proton will be transferred from the PIL to a base, though it must be noted that the pH is usually considered for aqueous solutions and may not be appropriate for the nonaqueous PILs. (b) Proposed doping mechanism in presence of protic ionic liquid. Reprinted with permission from ref. 129. Copyright 2013 American Chemical Society. (c) Molecular structure of *N*-butyl-*N*′-(4-pyridylheptyl)imidazolium bis(trifluoromethane)sulfonimide (BuPyIm-TFSI). (d) Molecular structure of BMPY-TFSI (e) evolution of photovoltaics parameters of the devices containing Spiro-OMeTAD doped with different concentration of BMPyTFSI and conventional dopant. These devices were kept in darkness and under humidity (>50% relative humidity) and were monitored continuously. Reprinted with permission from ref. 135. Copyright 2018 Elsevier.

Ionic liquids were one of the first non-Li/Co-based dopants assessed for HTLs in PSCs to eliminate byproducts in the HTL. In 2013, Abate *et al.* doped spiro-OMeTAD with protic ionic liquids (PIL) of varying pH (all with TFSI^–^ anions) (Fig. 7) leading to HTL conductivity increases by three orders of magnitude.^129^ They proposed a slightly altered doping mechanism with PIL (as compared to Scheme 1): first, the triarylamine nitrogen within spiro-OMeTAD is protonated by the PIL, and after electron injection from an adjacent pristine/neutral spiro-OMeTAD molecule, hydrogen gas is released, which allows for the radical cation to be trapped by a TFSI^–^ anion (Fig. 7). In contrast to LiTFSI/FK209-based doping, the redox chemistry with PIL is thermally activated and does not require oxygen exposure. Of the three ionic liquids, bis(trifluoromethanesulfonyl)imide (HTFSI)-doped spiro-OMeTAD HTLs in PSCs led to highest fill factor and PCE as compared to LiTFSI, 1-alkyl-3-methylimidazolium bis(trifluoromethane)sulfonimide (Himi-TFSI), or tetraethylammonium bis(trifluoromethane)sulfonimide (Et_4_N-TFSI)-based doping (Table 1, entry 1). They attributed these performance differences to reduced HTL charge-transport resistance. Notably, *t*BP reduced PSC performance in the presence of ionic liquid-doped HTL, which may be due to side reactions that prevent spiro-OMeTAD oxidation and/or reduce radical cations. With any *in situ* doping method, benign products/ions that minimally impact bulk properties are desired. In this respect, hydrogen gas production from HTFSI is an ideal scenario because the reaction byproducts do not remain in the HTL. However, a drawback of many of these protic ionic liquids is inherent ionic liquid hydrophilicity,^130^ and while a few follow-up studies have been reported,^131^,^132^ the potential impact(s) of HTFSI on PSC stability is(are) unclear because co-dopants were used alongside HTFSI in these studies (*i.e.*, Et_4_NTFSI, FK209 respectively). Nonetheless, Abate *et al.*^132^ assert the importance of HTM selection with respect to HTL dopant and PSC stability (Table 1, entry 8).

Zhang *et al.* utilized *N*-butyl-*N*′-(4-pyridylheptyl)imidazolium bis(trifluoromethane)sulfonimide (BuPylm-TFSI) (Fig. 7)^133^ as a lithium-free dopant, similar to a bromide analogue used for dye-sensitized solar cells (DSSC).^134^ The molecular design was threefold to leverage radical generation properties and overall HTL stability: spiro-OMeTAD doping *via* PIL, increased anion thermal stability with increased anion molecular weight, and potential interface passivation *via* the pyridyl group affixed to the alkyl chain (akin to *t*BP, and later seen in HTM design^63^). PSC with BuPylm-TFSI or LiTFSI-doped HTL generated comparable PCE (Table 1, entry 2). While the potential for increased thermal stability was purported, PSC performance at increased temperature was not reported. Additionally, BuPylm-TFSI synthesis requires pyrophoric diisopropylamide (LDA), so accessability may be limited to those with expertise.^134^ To the best of the authors's knowledge, this is a stand-alone report.

Recently, a commercially available aprotic ionic liquid, 1-butyl-3-methylpyridinium bis(trifluoromethane)sulfonimide (BMPyTFSI, Fig. 7), was incorporated into a spiro-OMeTAD-based HTL.^135^ This ionic liquid is similar to quaternary ammonium cation-based Et_4_N-TFSI utilized by Abate *et al.*^129^ except for the incorporation of the aromatic group, which increases the material's hydrophobicity. In contrast to PIL discussed previously, the HTL solutions with aprotic ionic liquids required oxygen exposure to generate spiro-OMeTAD radical cations before device fabrication and are not directly redox active. Interestingly, HTL conductivity increased from 4.9 × 10^–8^ S cm^–1^ (pristine spiro-OMeTAD) to 4.4 × 10^–6^ S cm^–1^ (7.8 mol% BMPyTFSI). This is in contrast to the reduced conductivity observed by Abate *et al.*^129^ with Et_4_N-TFSI dopant at similar concentrations (∼10 mol%). Nevertheless, devices with BMPyTFSI-doped spiro-OMeTAD performed similarly to devices with LiTFSI/FK209/*t*BP-doped HTL. FF and *V*_oc_ were slightly lower than controls due to higher HTL series resistance (Table 1, entry 27). Device stability was assessed over 200 days. Devices were unencapsulated and stored in a humidity chamber (∼50% relative humidity) in the dark and photovoltaic properties were periodically assessed. Devices with BMPyTFSI-doped HTL maintained up to 80% of initial PCE under these conditions, attributed to a *J*_sc_ drop, while control devices lost over 50% initial PCE (Fig. 7). HTL hydrophobicity was monitored over time, and water contact angle measurements remained constant (>90° *vs.* >78° control). While this aprotic ionic liquid clearly has superior hydrophobicity for improved PSC stability, the degradation mechanism(s) for performance loss is unclear at this time, particularly for devices under operational conditions.

### 3.2 Brønsted/Lewis-acids

**Fig. 8:**
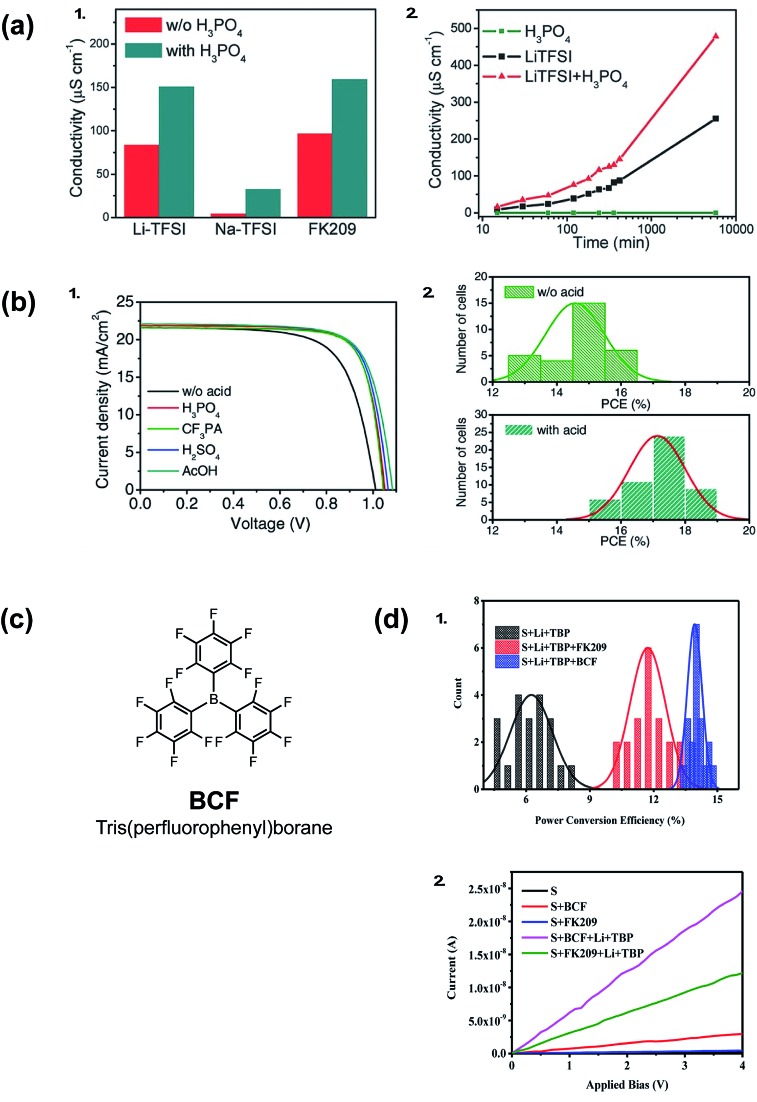
(a) (1) Conductivity of spiro-OMeTAD thin films with different dopants. (2) Conductivity of spiro-OMeTAD thin films as function of aging time, the films were stored in a dry air environment. (b) PSC performance with and without acid additives (spiro-OMeTAD is predoped with Li-TFSI and FK209 to achieve a better baseline performance). (1) Light *J*–*V* curves of solar cells with different acids. (2) Statistical PCE distribution of solar cells. Reprinted with permission from ref. 136. Copyright 2016 John Wiley and Sons. (c) Chemical structure of BCF (d) (1) Statistical histogram of PCE of 20 devices for each of the three kinds of PSCs. (2) Conductivity of the spiro-OMeTAD films with different p-type dopants. Reprinted with permission from ref. 137. Copyright 2017 American Chemical Society.

Acid doping is not limited to ionic liquids. In 2016, Li *et al.* show acid additives with a wide p*K*_a_ range, such as phosphoric acid, sulfuric acid, acetic acid, or (4-(trifluoromethyl)styryl)phosphonic acid, can improve the conductivity of spiro-OMeTAD films (Fig. 8). In conjunction with LiTFSI/FK209/*t*BP, the acid-doped systems result in improved *V*_oc_, fill factor, and reduced *J*–*V* hysteresis (Table 1, entry 14).^136^Fig. 8 exemplifies superior HTL properties with 10 mol% phosphoric acid: not only does HTL conductivity increase at a greater rate than with LiTFSI alone, but conductivity improvements are observed over many hours. Therefore, it can be inferred that radical spiro-OMeTAD cations are not appreciably reduced (quenched) in ambient conditions. Upon PSC integration, PCE increased from 15.2% to 17.6% when the HTL contained 10% phosphoric acid in addition to LiTFSI/FK209. In short, acid-doping eliminated the need for traditional device aging with ambient oxygen. Intriguingly, varying the acid strength/proton lability facilitated similar performance improvements. It was proposed that weak hydrogen bonding between the acid and spiro-OMeTAD may increase positive charge carriers, or acid additives may improve interfacial contact with the PAL to reduce recombination losses. Shelf-life stability of PSCs with and without phosphoric acid doping were not significantly different (Table 1, entry 14).

A Lewis acid, tris(pentafluorophenyl)borane (BCF), in conjunction with LiTFSI also improved conductivity and device efficiencies, as reported by Ye *et al.* in 2017 (Fig. 8).^137^ UV-Vis absorption spectroscopy and electron-pair spin resonance (EPR) spectroscopy affirm BCF's ability to generate radical spiro-OMeTAD^+^˙ species without a co-dopant. However, only in conjunction with LiTFSI or FK209 were improved HTL conductivies/PSC FF/PCEs observed (Table 1, entry 15), which suggests BCF is not sufficient to stabilize radical species. Improved performance was attributed to increased dopant solubility and reduced HTL roughness.

In summary, acid co-doping can improve PSC performance with traditionally-doped spiro-OMeTAD-based HTL, yet there is currently little known on PSC stability impacts with these additional additives. Ultimately, acid-doping for improved PSC performance could have greater utility if the hygroscopic co-dopants (LiTFSI, FK209) were replaced with alternative, non-hygroscopic dopants.

### 3.3 Metal-based salts

A wide variety of metal-based salts as dopants have been popular in addition to Li- and Co-based salts because they are cost-effective, commercially available, and can promote redox reactions with spiro-OMeTAD. Moreover, many metal-based dopants do not require co-dopants, like LiTFSI or FK209, for radical cation synthesis.

**Fig. 9:**
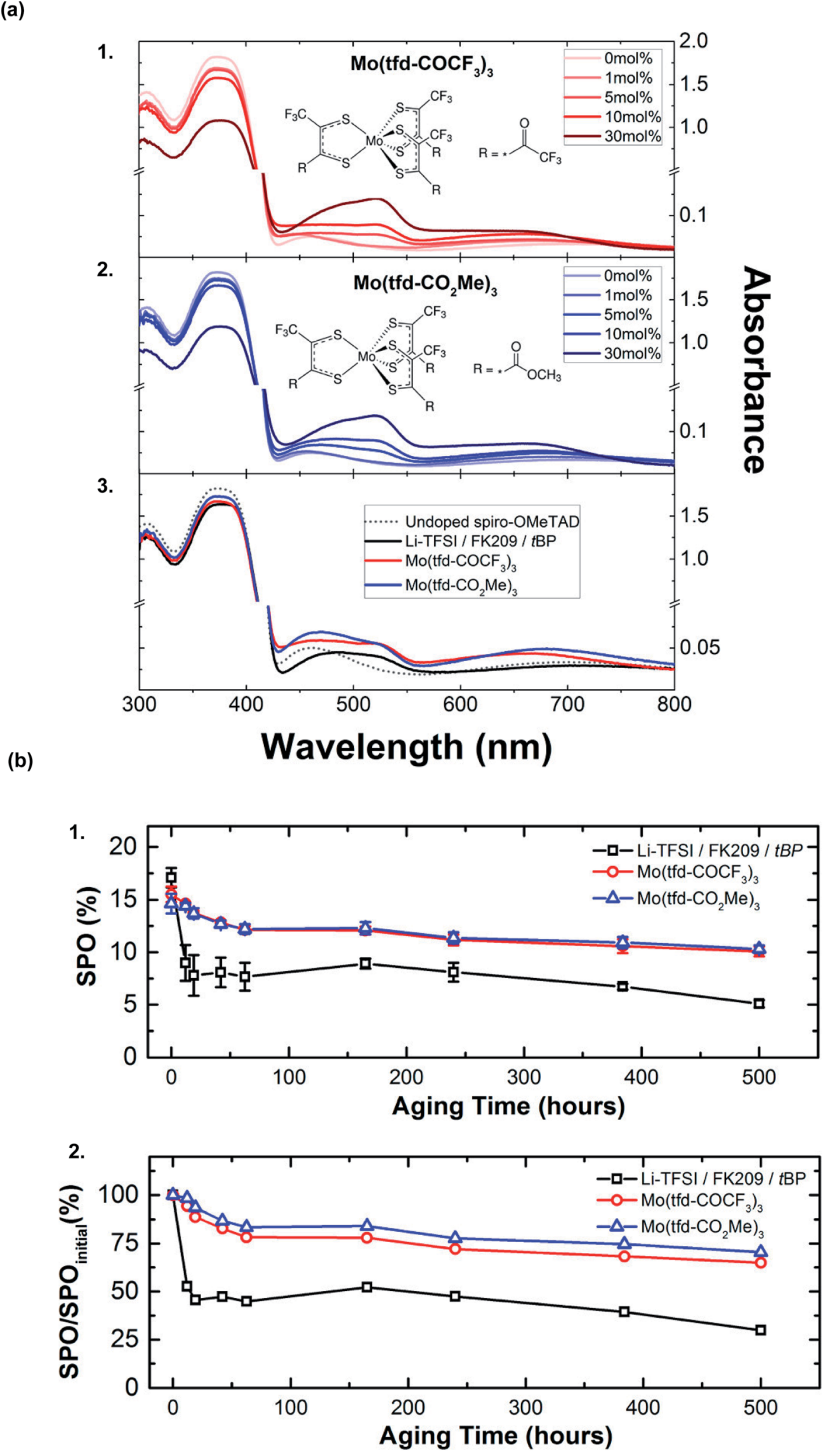
(a) Changes in the ultraviolet-visible (UV-Vis) absorption spectra of films of spiro-OMeTAD with the addition of (1) Mo(tfd-COCF_3_)_3_ and (2) Mo(tfd-CO_2_Me)_3_ to the casting solution, with their respective chemical structures shown as insets. Peaks at 520 and 690 nm can be assigned to oxidized spiro-OMeTAD (likely spiro-OMeTAD^+^, but spiro-OMeTAD^2+^ has an essentially identical spectrum), with minor contributions from Mo(dt)_3_^–^ absorptions. (3) Absorption spectra of pristine spiro-OMeTAD compared to the standard Li-TFSI/FK209/*t*BP-doped and molybdenum-doped spiro-OMeTAD. For both Mo(tfd-COCF_3_)_3_ and Mo(tfd-CO_2_Me)_3_ a molar concentration of 5% relative to spiro-OMeTAD was chosen. The molar concentrations of additives for the Li-doped reference with respect to spiro-OMeTAD are 50%, 3%, and 330% for Li-TFSI, FK209, and *t*BP, respectively. (b) Aging for 500 h at 85 °C in the dark at 30% humidity of nonencapsulated high-performance perovskite solar cells comparing Li-TFSI/FK209/*t*BP and Mo(dt)_3_-doped spiro-OMeTAD. (1) Average stabilized efficiencies of eight devices measured by holding the devices at their *J*–*V* determined maximum power point for 60 s with the corresponding standard deviations. (2) Average stabilized efficiencies over the initial stabilized power output. Within 24 h, lithium-doped devices deteriorate by 50% while both Mo(dt)_3_-doped devices sustain 70% of their initial performance after 500 h of aging. Reprinted with permission from ref. 140. Copyright 2017 American Chemical Society.

Molybdenum-based dopants are well-known and used to dope a number of p-type organic materials.^138^,^139^ Previously, molybdenum-based dopants have been successfully used for doping polymer-based HTLs, such as P3HT^138^ and PEDOT.^139^ With respect to small-molecule HTLs, solution-processable molybdenum tris(1-(methoxycarbonyl)-2-(trifluoromethyl)ethane-1,2-dithiolene) (Mo(tfd-CO_2_Me)_3_) and molybdenum tris(1-(trifluoroacetyl)-2-(trifluoromethyl)ethane-1,2-dithiolene) (Mo(tfd-COCF_3_)_3_) were assessed by Pellaroque *et al.*^140^ This is not commercially available, and dopant synthesis required three simple steps with moderate yields.^138^,^141^ Upon dopant addition, spiro-OMeTAD rapidly oxidized in a nitrogen atmosphere per UV-Vis absorption spectra (Fig. 9), in contrast to LiTFSI-based doping which requires oxygen. Additionally, lower dopant concentrations were required for improved thin-film conductivity – 5 mol% of either molybdenum-based dopant yielded a higher conductivity as compared to 30 mol% LiTFSI/FK209/*t*BP. Non-encapsulated devices (aged for 500 hours in the dark at 85 °C at 30% humidity) retained ∼70% PCE with either molybdenum tris(dithiolene)-doped HTLs (Fig. 9, Table 1, entry 16). Improved mechanistic understanding of PSC stability impacts at varying temperatures and humidity is needed (*e.g.*, molybdenum ion mobility under different aging conditions, such as under electrical bias). Based upon these findings, further dopant structure design (*e.g.*, dopant ligand tuning) may facilitate increased PSC performance alongside lifetime improvements.

Copper salts are another class of inexpensive, widely utilized p-type material. Copper salts^29^,^142^ and copper phthalocyanines^143^–^145^ have been used as HTLs for PSCs due to high conductivities, hydrophobicity, and low cost. However, the salts display low orthogonal solubility with the active layers, making solution-processing challenging. Furthermore, they can suffer from low efficiencies due to relatively higher recombination and poor FF, possibly resulting from phase changes.^143^,^145^,^146^

**Fig. 10:**
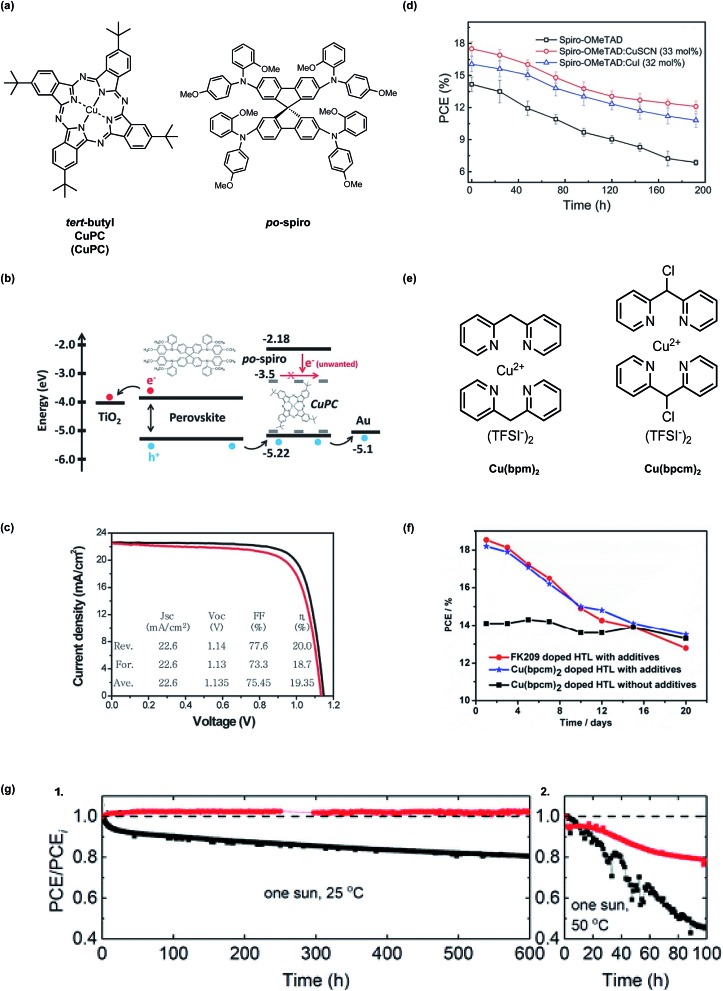
(a) Chemical structures of *tert*-butyl copper(ii) phthalocyanine (CuPC) and *po*-spiro (b) The schematic image for energy level alignment of the materials used in our devices and the proposed working mechanism of CuPC as a dopant in the *po*-spiro HTM layer. (c) *J*–*V* curve for the best device with a CuPC (4.8 wt%)-doped *po*-spiro HTM under reverse (black) and forward (red) scans. The inset shows the average values of the photovoltaic parameters obtained from *J*–*V* curves under both scans. Reprinted with permission from ref. 147. Copyright 2015 John Wiley and Sons. (d) Cell stabilities of PCE in spiro-OMeTAD, spiro-OMeTAD:CuSCN (33 mol%), and spiro-OMeTAD:CuI (32 mol%) based PSCs evaluated in the atmosphere at the room temperature. Reprinted with permission from ref. 148. Copyright 2016 John Wiley and Sons. (e) Chemical structures of bis[di(pyridin-2-yl)methane] copper(ii) bis[bis(trifluoromethyl-sulfonyl) imide] [Cu(bpm)_2_] and bis[2,2′-(chloromethylene)-dipyridine] copper(ii) bis[bis(trifluoromethylsulfonyl) imide] [Cu(bpcm)_2_]. (f) Stability of PSCs under ambient conditions (humidity 30–40% and temperature 20–25 °C), devices were kept in dark condition after test. Reprinted with permission from ref. 149. Copyright 2017 American Chemical Society and reuse under CC-BY-NC-ND. (g) Long-term stability of devices at (1) 25 °C and (2) 50 °C with respect to the dopant for spiro-MeOTAD in the presence of FK209. Black and red symbols represent Li-TFSI and Zn-TFSI_2_, respectively. PCE values were divided by the initial value (PCE_i_). The black horizontal dashed line of (a) is a guide line indicating 1.0. The devices were maintained at the mpp under one sun (100 mW cm^–2^) and a N_2_ atmosphere. Reproduced from ref. 150 with permission from The Royal Society of Chemistry.

In 2015, Seo *et al.*^147^ incorporated *tert*-butyl copper(ii) phthalocyanine (CuPC) to dope *N*^2^,*N*^2^′,*N*^7^,*N*^7^′-tetrakis(2-methoxyphenyl)-*N*^2^,*N*^2^′,*N*^7^,*N*^7^′-tetrakis(4-methoxyphenyl)-9,9′-spirobi[fluorene]-2,2′,7,7′-tetraamine (*po*-spiro), a spiro-OMeTAD analogue (Fig. 10). Mechanistically it is unclear as to how CuPC interacts with *po*-spiro, as no HTL film characterization was provided, but no co-dopants were required in addition to CuPC. In PSCs with CuPC dopant, PCE increased by >1% with CuPC (Fig. 10, Table 1, entry 6) due to reduced electron leakage at the interfaces, which was attributed to longer recombination transient photovoltage decay and favorable frontier molecular orbital energetic alignment.

Copper iodide (CuI) has been shown promote radical cation generation with LiTFSI.^151^ Soon after, both CuI and copper thiocyanate (CuSCN) were reported to act as p-type dopants for spiro-OMeTAD without LiTFSI.^148^ In dark stability assessment at ambient temperatures and humidity, CuSCN-doped spiro-OMeTAD maintained ∼80% of its initial PCE, outperforming devices with CuI or LiTFSI-doped HTLs (Summarized in Fig. 10 and Table 1, entry 9). Improved performance with CuSCN compared to CuI was attributed to favorable HTL morphology: reduced aggregation, crystallization, and pinhole formation was observed in CuSCN-doped HTL films. Even though spiro-based HTLs are amorphous, morphological changes induced by additives can have a tremendous impact on macroscopic properties. In this case, simply interchanging iodide for thiocyanate determined the final HTL film quality.

In 2017, bis[di(pyridin-2-yl)methane] copper(ii) bis[bis(trifluoromethyl-sulfonyl) imide] (Cu(bpm)_2_) and bis[2,2′-(chloromethylene)-dipyridine] copper(ii) bis[bis(trifluoromethylsulfonyl)imide] (Cu(bpcm)_2_) (Fig. 10),^149^ were assessed by Chen *et al.* These dopants were synthesized in 3 and 4 steps respectively with good yields, but the amine ligand synthesis required pyrophoric *n*-butyl lithium. The main difference between the two dopants is the chlorine incorporation in Cu(bpcm)_2_. Chlorine is inductively electron withdrawing, and this ligand tuning deepens the highest occupied molecular orbital (HOMO) level as compared to Cu(bpm)_2_, which, in turn, increases the oxidation driving force in the presence of spiro-OMeTAD. Cu(bpcm)_2_ readily oxidized spiro-OMeTAD at approximately 75% yield per UV-Vis absorption characterization without oxygen exposure, approaching quantitative conversion that traditional dopants do not consistently yield. Unfortunately, preliminary stability assessments in the dark at 30–40% relative humidity at 20–25 °C only small lifetime improvements were observed, and the degradation mechanism(s) is (are) unclear (Table 1, entry 17). While Cu(bpcm)_2_ shows the highest doping efficiency/radical cation generation per UV-Vis, and corresponding increase in HTL conductivity, devices with Cu(bpcm)_2_-doped HTLs do not perform as well as those doped with Cu(bpcm)_2_/LiTFSI or FK209/LiTFSI (Fig. 10). After 20 days, all devices in the stability study, with and without LiTFSI, retain ∼75% PCE.

Recently, Zhang *et al.*^152^ investigated hexafluorophosphate (PF_6_^–^) copper complexes. Three complexes with different redox potentials were compared. One dopant, (bis[di(pyridin-2-yl)methane] copper(ii) bis[hexafluorophosphate], JQ1), was identical to that of Cu(bpm)_2_ reported by Chen *et al.*^149^ with exception of the anion. Interestingly, the bis[di(pyridin-2-yl)methane] copper(ii) TFSI^–^-based copper complex dopant was deemed ineffective and was not integrated into PSCs^149^ yet Zhang *et al.* found the bis[di(pyridin-2-yl)methane] copper(ii) PF_6_^–^-doped HTL led to the highest performing PSCs (Table 1, entry 28).^152^ While the dopants presented in this review predominantly use TFSI^–^ as an inert counteranion, this particular example showcases the counterion's less understood importance.

In addition to dopant concentration optimization for best device-level performance, Zhang *et al.*^152^ also considered (1) doping efficiency (ratio copper salt to spiro-OMeTAD^+^˙ cations), (2) dopant redox potential, and (3) “ideal” spiro-OMeTAD^+^˙ concentrations. They hypothesize that higher radical cation concentrations lead to increased charge-recombination at the PAL/HTL interface and lower *V*_oc_, and in the context of the copper-based dopants, they found 1 mol% spiro-OMeTAD^+^˙ correlated with best device-level performance regardless of copper ligand identity. While these findings may not be directly applicable to all doping systems, it is one of the few studies with a focus on dopant optimization, subsequent radical cation concentration, and interface dynamics. While devices with JQ1-doped HTLs displayed best initial performance, for the stability study, the HTL was doped with bis[2-methyl-6-(6-methylpyridin-2-yl)pyridine] copper(ii) bis[hexafluorophosphate] (JQ3) instead of JQ1. A 1 nm Al_2_O_3_ interface was required to suppress recombination at the PAL/HTL interface, improve initial PSC PCE and stability of devices stored in ambient conditions in the dark at 25 °C and 50% relative humidity (Table 1, entry 28). The required buffer layer for improved PSC stability suggests water ingress is an important degradation pathway to mitigate for devices with JQ3-doped HTLs.

Overall, copper-based metal organic complexes and salts are noteworthy candidates due to their low cost and wide availability, but again, more comprehensive stability and lifetime assessment are needed.^17^

The first zinc-based salt was reported as an effective dopant in HTL for PSC. Zinc bis(trifluoromethanesulfonyl)imide (Zn(TFSI)_2_) was used to dope spiro-OMeTAD, and increased HTL hole mobility by an order of magnitude as compared to LiTFSI (with TFSI^–^ anion concentration constant).^150^ Upon device integration, devices with Zn(TFSI)_2_-doped HTL generated increased PCE (21.52% *vs.* 19.48% with LiTFSI) resulting from *V*_oc_ and FF gains (Table 1, entry 26). The best PSC performance required FK209 as co-dopant. In one stability assessment, PSC were subjected to one sun at 25 °C at maximum power point under a N_2_ atmosphere and HTLs were doped with Zn(TFSI)_2_ or LiTFSI and FK209. Initial efficiencies are >20%, but over the course of 600 hours, the devices with Zn-doped HTL maintained 100% initial PCE, while devices with Li-doped HTL maintained 80%. After 100 hours at 50 °C, PSCs with Zn-doped HTL maintained roughly 80% initial PCE, while PSC with Li-doped HTL maintained roughly 45% initial PCE (Fig. 10). In a shelf life assessment, devices were unencapsulated and stored in the dark at room temperature in between device parameter measurements, and FK209 was not added as a co-dopant. Over 800 hours in a dry atmosphere, PCEs observed in devices with Zn-doped and Li-doped HTLs remain relatively constant. When the relative humidity is increased to 40%, FF and PCE drop in both cases, but a slower rate of decay is observed in devices with Zn-doped HTL. Unencapsulated PSC stability with FK209 is not reported. Overall, a device performance and stability tradeoff is not observed with Zn-doped HTLs when devices are “perfectly encapsulated” (in a N_2_ atmosphere).

Generally, metal-based dopants are attractive because they lead to high performing PSC with low-cost materials. With regard to other metals, iridium and iron salts have been reported in standalone reports. An IrCp*Cl(PyPyz)[TFSI]-based doping system^153^ showed superior stability (96% PCE retention over 3 months in the dark in ambient conditions) over control devices (Table 1, entry 5). Benzoyl peroxide^154^ and iron-based complexes^155^,^156^ also successfully dope spiro-OMeTAD with LiTFSI co-dopant for efficiency improvements (Table 1, entries 19, 23, and 24 respectively). A lithium-ion endohedral fullerene (Li^+^@C_60_) was also recently reported (Table 1, entry 25).^157^ Unencapsulated devices under constant illumination produced power as much as 10 times longer than the control devices.

It is challenging to directly compare stability since conditions vary, but with regard to both high performance and low cost, Zn(TFSI)_2_-based doping is one of the most promising systems, as the dopant is low cost and PSC showed excellent stability in a variety of conditions. The most pressing concerns that remain include (1) inherent hydrophilicity (2) need for co-doping (LiTFSI, FK209), (3) potential for ion migration as observed with a number of metal electrodes (*e.g.*, Au, Ag, Li^+^, Na^+^, K^+^),^22^,^158^,^159^ and (4) the potential for redox reaction occurring between metal and the perovskite itself.^160^ If these factors can be addressed, metal-based dopants could be a cost-effective solution for both highly efficient and stable PSC.

### 3.4 Oxidized radical cation salts

Reduced oxygen exposure, hygroscopic lithium elimination, and dopant concentration consistency are important for batch to batch consistency and PSC lifetime. Ionic liquids, such as HTFSI, fulfill the first two criteria. Oxidized radical cation salts fulfill all of these requirements and can be engineered with greater hydrophobicity than ionic liquids.

**Fig. 11:**
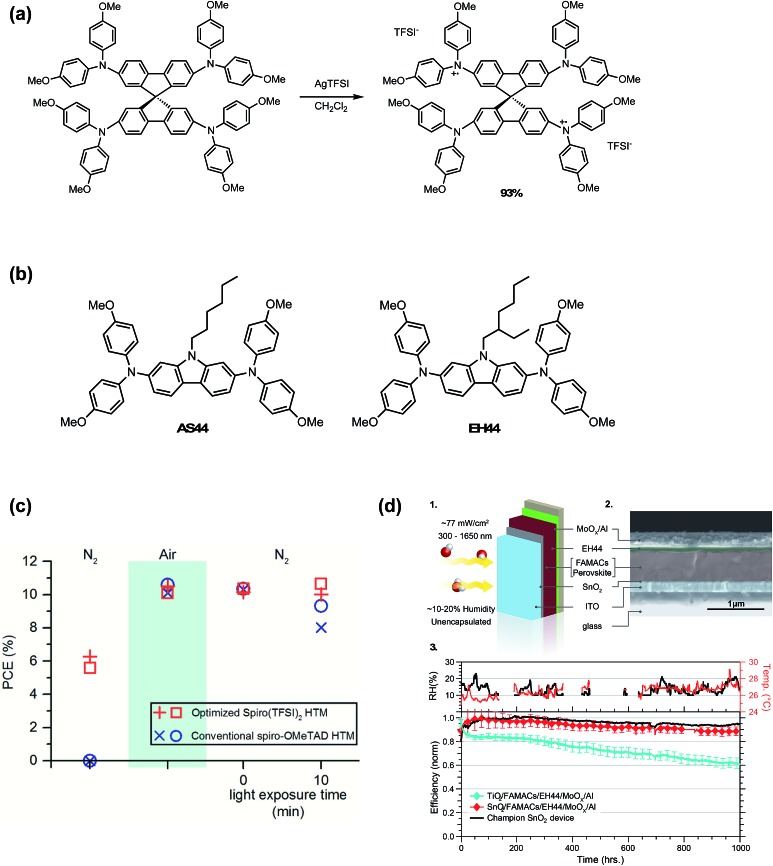
(a) Synthesis of spiro(TFSI)_2_. Reprinted with permission from ref. 98. Copyright 2014 American Chemical Society. (b) Chemical structures of AS44 and EH44. (c) Power conversion efficiencies of PSCs with either a 12 mol% spiro(TFSI)_2_ HTM or conventional spiro-OMeTAD HTM under three consecutive environmental testing conditions: (1) nitrogen atmosphere, never exposed to air; (2) exposed to air; (3) reintroduced to a nitrogen atmosphere after being exposed to air. Upon reintroduction to a nitrogen atmosphere, devices with spiro(TFSI)_2_ maintained greater than 98% of initial efficiencies after 10 min of illumination compared to less than 90% for devices without. Reprinted with permission from ref. 98. Copyright 2014 American Chemical Society. (d) Operational stability of ETL/FAMACs/EH44/MoO_*x*_/Al devices in ambient. (1) Schematic that shows the test conditions for the devices. Yellow arrows represent illumination and red and white spheres the oxygen and hydrogen atoms, respectively. (2) SEM cross-section image of a SnO_2_/FAMACs/EH44/MoO_*x*_/Al device. (3) The ambient relative humidity and room temperature were monitored during the course of the experiments (top). Normalized average efficiency obtained from current–voltage scans over time (bottom) for devices of the type ETL/FAMACs/EH44/MoO_*x*_/Al in which the ETL layer is either TiO_2_ (four devices) or SnO_2_ (15 devices). The initial 1 sun performance of these devices is shown in Table 3 in ref. 127. The champion stability for a SnO_2_/FAMACs/EH44/MoO_*x*_/Al device is shown as a black line. The devices were held under a constant resistive load (510 Ω) and actively cooled using a circulating bath set to 20 °C with the device surface measuring approximately 30 °C. Reprinted with permission from ref. 127. Copyright 2018 Springer Nature.

To eliminate byproducts from *in situ* radical cation generation, Nguyen *et al.*^98^ prepared a spiro-OMeTAD TFSI (spiro(TFSI)_2_) oxidized salt dopant in one step and high yields from silver bis(trifluoromethane-sulfonyl)imide (AgTFSI) (Fig. 11). Compared to LiTFSI, spiro(TFSI)_2_ displayed lower solubility, and required heating precursor solution to 70 °C. Since spiro-OMeTAD had already been oxidized in advance of HTL precursor preparation, spiro(TFSI)_2_-doped devices yielded functional devices without oxygen exposure. After air exposure and return to nitrogen atmosphere, devices fabricated with spiro(TFSI)_2_ achieved comparable efficiencies to controls yet retained stable performance over 10 minutes of illumination (Fig. 11 and Table 1, entry 3). In contrast to synthesizing and purifying the HTM oxidized salt, AgTFSI has also been used for *in situ* doping of HTL solutions^161^ and for Ag-HTM-based HTLs.^162^ However, any remaining Ag^+^ in HTL would likely hinder stability due to AgI formation observed in devices employing Ag electrodes.^163^

In 2016, Leijtens *et al.*^164^ assessed multiple oxidized salts for use as dopants. To determine the influence of the anion on conductivity, spiro(TFSI) and spiro(SbCl_6_) were prepared. At equivalent mole%, TFSI^–^ affords higher conductivities as compared to SbCl_6_^–^, likely due to the more weakly coordinating nature of the TFSI^–^ anion. Additionally, other non-spiro-OMeTAD synthetically facile and hydrophobic HTMs (Fig. 11), namely 9-hexyl-*N*_2_,*N*_2_,*N*_7_,*N*_7_-tetrakis(4-methoxyphenyl)-9*H*-carbazole-2,7-diamine (AS44) and 9-(2-ethylhexyl)-*N*_2_,*N*_2_,*N*_7_,*N*_7_-tetrakis(4-methoxyphenyl)-9*H*-carbazole-2,7-diamine (EH44) were evaluated,^164^ doped with their respective oxidized TFSI^–^ salts (AS44-ox and EH44-ox, respectively). AS44/AS44-ox and EH44/EH44-ox performed similarly to Li-doped spiro-OMeTAD (Table 1, entry 10), yet had higher solubility in precursor solutions. Of the three HTL systems in this study, EH44/EH44-ox HTL demonstrated superior hydrophobicity. While visible yellow PbI_2_ crystallites formed nearly instantaneously when spiro-OMeTAD- and AS44-based devices were submerged in water, no visible PbI_2_ formation was observed when EH44-based devices were briefly submerged. In 2018, further stability studies by Christians *et al.*^127^ demonstrate superior operational lifetime and stability of the EH44/EH44-ox system as compared to spiro-OMeTAD/LiTFSI and spiro-OMeTAD/spiro-ox. After perovskite active layer, metal electrode, and ETL optimization, the champion unencapsulated EH44/EH44-ox device maintained 94% PCE over 1000 hours of continuous operation at ambient temperature and humidity (Fig. 11). However, the PCEs of devices doped with EH44-ox were generally lower than those of Li-doped HTLs (<16%) (Table 1, entry 22). Additionally, at elevated temperatures, significant degradation is observed, likely due to low glass transition temperature (*T*_g_) of EH44.

Ultimately, for widespread use of metal-free, oxidized radical cation salts as dopants, HTL thermal robustness and overall efficiency must be addressed. Importantly, dopant structure, in addition to device stack optimization (*i.e.*, PAL, ETL, contacts), are vital to mitigate materials-level degradation pathways observed in radical cation-doped HTL.^165^,^166^

### 3.5 Tetracyanoquinodimethane (TCNQ) derivatives

A drawback of the systems discussed thus far is that they are limited to solution-processing. While solution methods are certainly one attractive route for PSC scale-up,^167^ tetracyanoquinodimethane (TCNQ) and TCNQ derivatives are dopants compatible with both vacuum and solution-processing. TCNQ is a known electron acceptor capable for forming charge-transfer complexes for improved optoelectronic properties,^168^ and fluorinated TCNQ derivatives have successfully doped spiro-OMeTAD-based HTLs, and improved HTL hydrophobicity *via* hydrophobic fluorine atom incorporation and metal cation elimination.

**Fig. 12:**
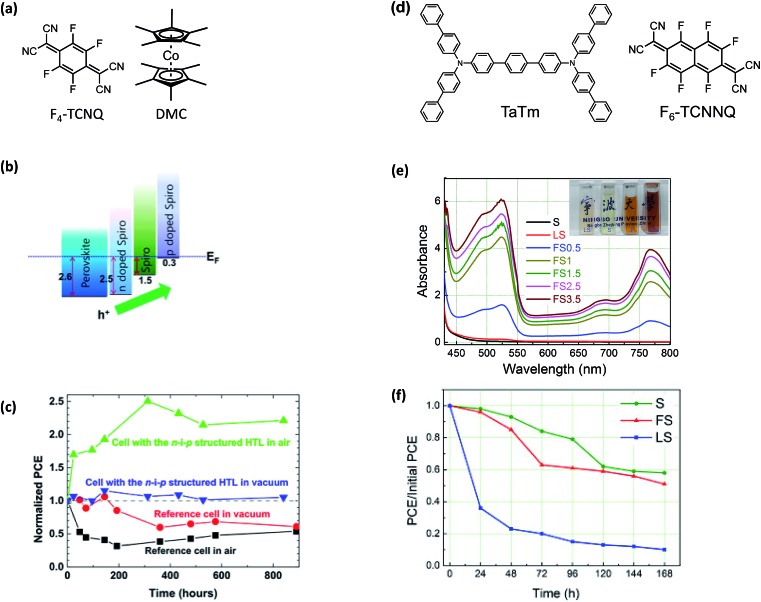
(a) Chemical structures of F4-TCNQ and DMC (b) energy level alignment diagram extracted from the UPS results. (c) The average value of PCE for the freshly-prepared solar cells is used as the normalization reference value (*i.e.* for the four types of samples, their initial PCE values are all normalized to (1)). All measurements have been done after pre-illuminating the solar cells for 40 s under the open circuit condition. The *j*–*V* scan was performed from 1.1 V to 0 V at a scan rate of 0.2 V s^–1^. After 800 h, the reference samples stored in air and in vacuum showed 50–60% of their initial efficiencies. However, the two cells with the n–i–p structure HTL did not show any sign of degradation after 800 h under both storage conditions. Reprinted with permission from ref. 169. Copyright 2015 Springer Nature and reuse under CC-BY 4.0 (d) chemical structures of TaTm and F6-TCNNQ. (e) UV-Vis absorption spectra of spiro-MeOTAD (S), mixture of spiro-MeOTAD and Li-TFSI (LS) and mixture of spiro-MeOTAD and F4-TCNQ (FS) with different molar ratio (0.5%, 1%, 1.5%, 2.5%, 3.5%) in chlorobenzene. The inset gives the images of cuvettes filled with LS, S, F, FS chlorobenzene solutions, respectively. (f) Stability investigation of the unencapsulated PH PVKSCs with different HTLs. S represents pristine spiro-OMeTAD, FS represents spiro-OMeTAD + 1% F4-TCNQ, and LS represents spiro-OMeTAD + LiTFSI + *t*BP. Reprinted with permission from ref. 172. Copyright 2016 Elsevier.

In 2015, Qi and coworkers reported improved stability and reduced energetic barriers for charge extraction *via* a triple-layer HTL.^169^ The triple-layer HTL used in this work consisted of an (a) n-doped spiro-OMeTAD (dopant: decamethylcobaltocene, DMC), (b) pristine spiro-OMeTAD, and (c) p-doped spiro-OMeTAD (dopant: 2,3,5,6-tetrafluoro-7,7,8,8-tetracyanoquinodimethane, F4-TCNQ) (Fig. 12a). Each HTL layer (n-doped, intrinsic, and p-doped spiro-OMeTAD) was sequentially vacuum deposited onto the PAL (Fig. 12b). Upon operation, device efficiencies nearly double over hundreds of hours of air exposure which is attributed to possible dopant redistribution^170^ (Fig. 12 and Table 1, entry 7).

Another TCNQ derivative, 2,2′-(perfluoronaphthalene-2,6-diylidene)dimalononitrile (F6-TCNNQ), successfully doped a non-spiro-OMeTAD HTM, *N*^4^,*N*^4^,*N*^4^″,*N*^4^″-tetra([1,1′-biphenyl]-4-yl)-[1,1′:4′,1″-terphenyl]-4,4″-diamine (TaTm).^171^ TaTm was evaporated onto the PAL (*c.a.* 10 nm), and then F6-TCNNQ was co-evaporated with TaTm (*c.a.* 40 nm) for fully vacuum-deposited devices with high FF (79.8%) (Table 1, entry 18). Preliminary stability assessment were conducted under constant illumination at short circuit conditions and no temperature control, and n–i–p devices maintained ∼75% initial PCE. The loss in initial performance was attributed to loss in FF.

In addition to vacuum deposition techniques, TCNQ dopants are also soluble in organic solvents, like chlorobenzene, and can be integrated into fully solution-processed devices as reported by Huang *et al.* with F4-TCNQ.^172^ The doping efficiency of F4-TCNQ in spiro-OMeTAD was measured by UV-Vis absorption spectroscopy (Fig. 12e). While doping was achieved, the initial PCE of devices containing the F4-TCNQ-doped spiro-OMeTAD were somewhat lower than control devices with LiTFSI-doped spiro-OMeTAD (10.59% *vs.* 12.66%) (Table 1, entry 12). Despite the initial loss of efficiency, F4-TCNQ-doped devices showed similar stability to devices made with neat spiro-OMeTAD and dramatically better stability than those with LiTFSI-doped spiro-OMeTAD, demonstrating that F4-TCNQ doping does not facilitate degradation in the same way as LiTFSI doping. In contrast, Song *et al.* incorporated F4-TCNQ as an interfacial layer for reduced carrier recombination and improved efficiencies *via* halogen bonding passivation and interface doping (Table 1, entry 11).^173^ After 960 hours dark storage at room temperature without encapsulation, more than 60% of initial PCE was retained with F4-TCNQ interlayer and LiTFSI doped spiro-OMeTAD HTL.

Overall, the use of TCNQ-based derivatives is a promising doping technique as it allows for a non-hygroscopic additive using either solution or vacuum-processing, but the majority of examples still have the same limitation: lowered device efficiencies as a tradeoff for stability gains.

### 3.6 Additional chemical doping schemes

**Fig. 13:**
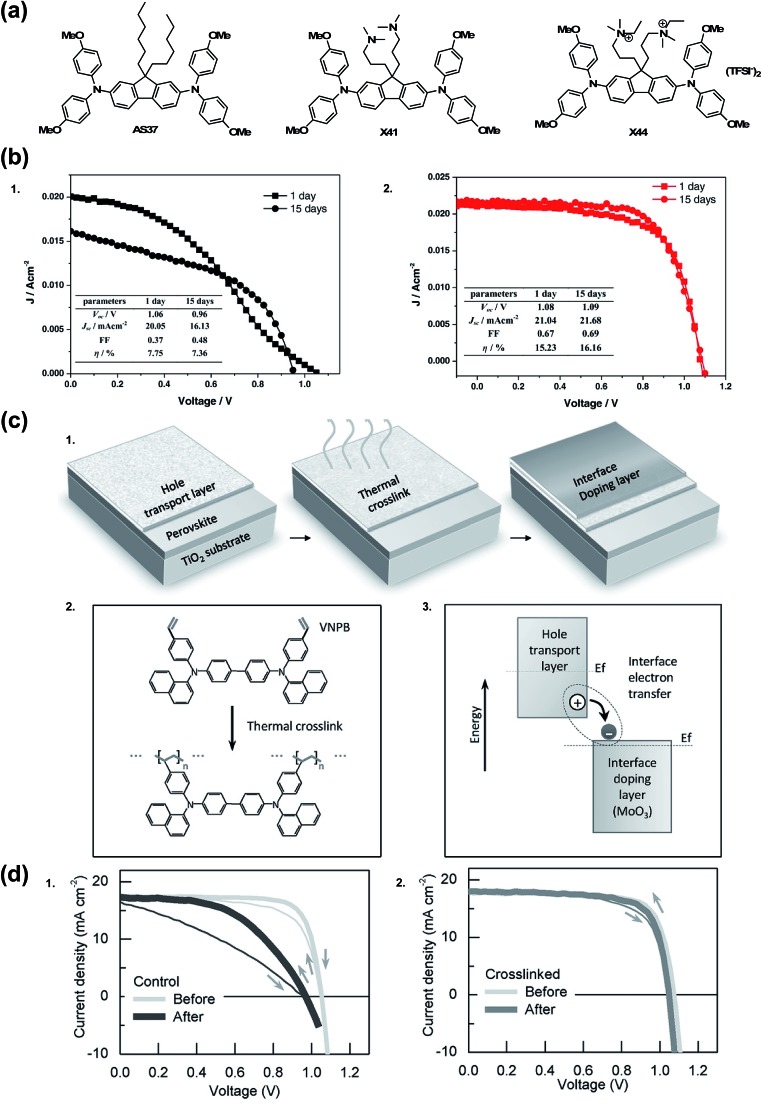
(a) Chemical structures of HTMs: AS37, X41, and X44. (b) Long-term stability test of the PSCs in a controlled humidity (<20%) in dark without encapsulation after 15 d for (1) AS37 and (2) X44. Reprinted with permission from ref. 174. Copyright 2017 John Wiley and Sons. (c) Hole extraction contact employing material crosslinking and interface doping. (1) Two-step scheme to form the insoluble and thermally stable hole extraction contact. In the first step, the organic hole transport layer (HTL) is deposited and then thermally crosslinked; in the second step, an interface doping layer is simply deposited atop the HTL and doping is achieved *via* the interface charge transfer. (2) Details of the thermal crosslinking process: double bonds in styrene groups in the hole transport layer (VNPB) are opened and then crosslinked *via* an addition reaction, thereby forming an insoluble, thermally stable film. (3) Schematic of interface doping: ground-state electron transfer occurs from the hole transport layer, having low ionization-energy, to the interface with the high electron-affinity material, in this case transition metal oxide MoO_3_, thereby enhancing the hole carrier density throughout the thin HTL. Evolution of performance, morphology, and material under external stress. (d) (1) The performance of devices using spiro-MeOTAD as the hole-extraction contact tested at room temperature (light gray) and after a 110 °C burn-in test (dark gray) [in the burn-in test, devices are annealed at 110 °C for 1 h in an N_2_ environment and tested after cooling down to room temperature]. (2) The performance of devices using VNPB-MoO_3_ tested at room temperature (light gray) and after 110 °C burn-in process (dark gray). Reprinted with permission from ref. 175. Copyright 2016 John Wiley and Sons.

There are a few noteworthy doped OSM HTL systems that do not fit into any of the previous categories. In 2015, Zhang *et al.*^174^ sought to simplify the HTL solution by incorporating TFSI^–^ anions into the chemical structure of the HTM (Fig. 13). It is of note that quaternary ammonium ions with TFSI^–^ counterions are present in 3,3′-(2,7-bis(bis(4-methoxyphenyl)amino)-9*H*-fluorene-9,9-diyl)bis(*N*-ethyl-*N*,*N*-dimethylpropan-1-aminium) bis(trifluoromethanesulfonyl)imide (X44), not radical triarylamine species, as presented previously.^129^,^135^ Even without radical cations, upon PSC integration, X44 showed superior hole conductivity as compared to AS37 (ref. 164) and 3,3′-(2,7-dibromo-9*H*-fluorene-9,9-diyl)bis(*N*,*N*-dimethylpropan-1-amine) (X41). Interestingly, while the conductivities of AS37 and X41 were comparable, devices with X41 as HTL did not function. On the other hand, devices with X44 as HTL showed good device efficiency and stability at maximum power point in the dark over 15 days (PCE 16.2% after aging from initial 15.2%) as compared to AS37 (PCE 7.8% after aging from initial 7.4%) (Table 1, entry 21).^174^ In a separate stability test, after 1 day of continuous light soaking, devices with X44-based HTL ∼60% initial efficiency was maintained, but stability suffered at elevated temperatures (>70 °C).

Remote doping is an alternative strategy for increasing hole carrier density. Xu *et al.* deposited MoO_3_ on undoped, cross-linked *N*^4^,*N*^4^′-di(naphthalen-1-yl)-*N*^4^,*N*^4^′-bis(4-vinylphenyl)biphenyl-4,4′-diamine (VNPB).^175^ The precise doping mechanism is unclear with certain metal oxides, like MoO_3_, as its energetic properties rapidly change in the presence of oxygen and metal electrode selection.^176^ Xu *et al.* demonstrated charge-transfer complex generation at the interface, and that free hole density increases because metal oxide accepts electron(s) from the HTM at the HTM/MoO_3_ interface. To summarize, the MoO_3_ dopes VNPB. Critically, the cross-linked VNPB prevented pinhole formation that would lead to MoO_3_ reacting with the active layer at the interface.^177^ Encapsulated devices employing VNPB/MoO_3_ HTL showed little change in FF as compared to spiro-OMeTAD/LiTFSI control after 1 hour at 110 °C in N_2_ (Fig. 13 and Table 1, entry 13). After dark storage in 70% relative humidity, decomposition is minimal as monitored by PbI_2_ emergence *via* XRD (but device performance was not reported). To the best of the author's knowledge, this is the only explicit report of remote doping for an HTL system in the PSC literature.

## 4. Summary and outlook

**Fig. 14:**
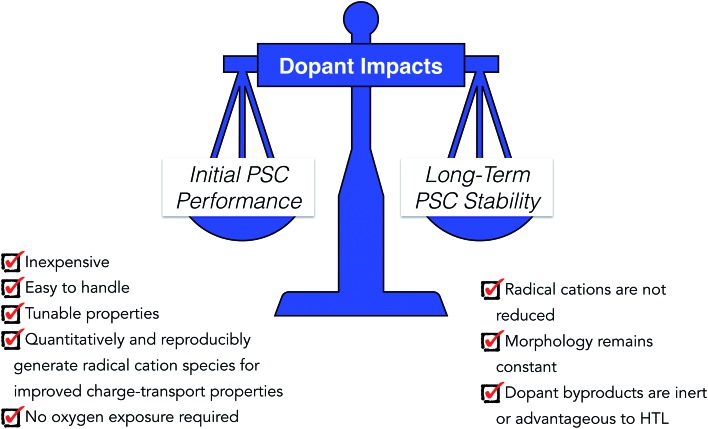
Proposed properties of ideal HTL dopants for high performing and stable PSCs.

Moving away from spiro-OMeTAD with LiTFSI/FK209/*t*BP is almost certainly required to meet hybrid organic/inorganic PSC stability goals. As discussed in this work, and compiled in Table 1, there are a number of promising doping strategies for small molecule-based HTLs for n–i–p architecture PSCs. Nevertheless, all of the methods discussed herein have shortcomings to overcome, some of which are perhaps insurmountable. First, many of the alternative dopant strategies highlighted in this review still utilize hygroscopic LiTFSI/FK209, making it likely that they will ultimately suffer similar stability issues as conventional spiro-OMeTAD-based devices even if some stability gains are achieved. Nevertheless, there are a few standout systems which we believe are interesting candidates for continued investigation. The oxidized radical cation salts as dopants, such as spiro(TFSI)_2_ (ref. 98) and EH44-ox,^127^,^164^ effectively eliminate metals and other byproducts of *in situ* doping strategies from the HTL. More specifically, the EH44/EH44-ox system shows very promising stability,^127^ yet still suffers from degradation under high temperature and humidity, likely at least in part a result of its low glass transition temperature. Metal-based dopants are generally inexpensive and generate efficient PSCs, but stability often suffers. Ultimately, widespread implementation of new dopants on the scale of LiTFSI/FK209 will depend not only on long-term performance but a number of factors, such as synthetic ease, cost, and fabrication compatibility (summarized in Fig. 14).

With respect to dopants for small-molecule HTLs, there are a number of imminent research needs that we believe will support the greater goals in the perovskite community regarding PSC stability and lifetime. We propose the following as clear research aims for concentrated efforts, analogous to efforts seen in stabilizing active layers, ETLs, *etc*. While many dopants *can* oxidize spiro-OMeTAD, improving charge-transport properties and PCE, there can be stark durability differences between doping schemes, even though the majority HTL constituent is composed on the same molecular matrix: spiro-OMeTAD. An increased understanding dopant-induced degradation mechanisms is required. As part of these experiments, operational cell-level PSC studies which probe operational stability will certainly be required over and above simple material stability or hydrophobicity questions. These studies will propel improved rational design and help to answer questions such as: are these chemical-induced degradation mechanisms, or morphological failures (*e.g.*, dopant migration, pinhole formation, *etc.*)? Can we design or modify materials to impede these degradation pathways? Which dopant schemes are the most inert?

Moreover, much of what is known is related to spiro-OMeTAD doping, yet there are hundreds of alternative HTMs already reported in the literature and an effectively infinite number yet to be explored or even synthesized. If organics are to be eventually used in commercial PSC modules these will likely be doped to improve transport properties, so we must therefore search for dopant and HTM design motifs. Synergistic efforts among HTM and dopant design will be necessary if they are to contribute to stable *and* efficient PSCs and ultimately to clean, inexpensive electricity generation.

## Conflicts of interest

There are no conflicts to declare.
